# The Role of Language Aspects in the Assessment of Cognitive and Developmental Functions in Children: An Analysis of the Intelligence and Development Scales–2

**DOI:** 10.1177/10731911251315027

**Published:** 2025-02-03

**Authors:** Salome D. Odermatt, Silvia Grieder, Florine Schweizer, Anette Bünger, Alexander Grob

**Affiliations:** 1 University of Basel, Switzerland

**Keywords:** language abilities, assessment, cognitive functions, developmental functions, Intelligence and Development Scales–2, multilingual children, bilingual children

## Abstract

The assessment of multilingual participants is challenging, as, for example, proficiency in the test language may interfere with test performance. We examined whether different language aspects (i.e., receptive and expressive language abilities, bi/multilingualism) contribute independently to the variance in scores on cognitive and developmental functions of the Intelligence and Development Scales–2 (IDS-2). The sample comprised 826 children aged 5 to 10 years living in German-speaking regions. Hierarchical regression analyses revealed that receptive language ability was significantly associated with almost all IDS-2 scores. Expressive language ability explained little additional variance, except for the intelligence composites, Verbal Reasoning (including subtests), and the basic skills subtests. Bi/multilingualism explained variance above language abilities only in subtests of Verbal Reasoning and verbal Long-Term Memory. Findings highlight the importance of considering language proficiency, particularly in tasks with high verbal demands, when assessing cognitive and developmental functions with the IDS-2 in participants at risk for linguistic disadvantages.

Assessments of individuals’ cognitive abilities and developmental domains play a crucial role in a comprehensive evaluation in clinicians’ daily practice. For example, performance on psychometric tests provides information about individuals’ strengths and difficulties and forms the groundwork for the development of clinical hypotheses and intervention measures (Flanagan & Harrison, 2012). Yet, participants’ test performance can be compromised by factors other than the examined construct, including aspects of measurement and participant characteristics, such as individuals’ level of proficiency in the test language as well as bilingualism and multilingualism (hereafter denoted: bi/multilingualism)—meaning the ability to understand and speak two or more languages (American Educational Research Association et al., 2014; American Psychological Association, 2015). This is particularly relevant as worldwide migration rates have been increasing over the past decades (United Nations, 2022). For instance, in German-speaking countries, the national censuses have identified approximately 29% of the German (German Federal Statistical Office, 2023), 39% of the Swiss (Swiss Federal Statistical Office, 2022a), and 25% of the Austrian (Austrian Federal Statistical Office, 2022) population as currently having a migration background.

Because of this relatively high percentage of individuals with a migration background, the number of children in schools and in psychological services who need educational assessment but whose native language is not the test language of the region in which they are living has consequently increased. Furthermore, in some cases, they may also have a different cultural background. Thus, the accurate assessment of these individuals’ abilities may be hampered, as, for example, bilingual and multilingual individuals often have insufficient knowledge of the test language, such as lower levels of language comprehension and grammar and smaller vocabularies in each of their languages (Bialystok et al., 2010; Hoff et al., 2012; Reich et al., 2002). Therefore, language issues may arise when conducting standardized tests (e.g., Guthke & Wiedl, 1996; Ortiz, 2019). Although there may also be cultural aspects that are associated with assessment in this context, such as previous experience with the content and material of the items or degree of acculturation (e.g., Guthke & Wiedl, 1996), in this study, we focused on the relative importance of language aspects for participants’ test performance.

In particular, individuals with linguistically diverse backgrounds may have difficulties understanding verbal instructions or, if required, giving verbal responses during the test administration (Cormier et al., 2022; Weiss et al., 2006). These constraints depend on the verbal demands a test places on the participant with respect to complexity, length, and verbosity of instructions (Cormier et al., 2011), response options, as well as the proportion of verbal components within the tasks (Flanagan & Ortiz, 2001). Specifically, children who are bilingual or have a migration background tend to exhibit lower test scores especially in tasks that are more verbal dependent (Hagmann-von Arx et al., 2013; Schweizer et al., 2021). As a result, these children might not show their full potential, which bears the danger of biased test results and in consequence may lead to an underestimation of their true abilities (Hagmann-von Arx et al., 2013). In particular, the abilities of younger children who enter the school system may be underestimated, as attending formal educational institutions enhances the development of language proficiency (Grob et al., 2014). This is critical, as an underestimation of abilities, such as intelligence, can lead to possible negative consequences regarding the children’s future school career and long-term development (Calero et al., 2013; Goldstein et al., 2015; Hessels, 1997; Klingner et al., 2007; Sullivan, 2011).

Thus, it is essential to evaluate the contribution of participants’ proficiency in the test language and bi/multilingualism to their performance on standardized assessments, to identify possible participant characteristics that could compromise the validity of the test score interpretation (see also fairness in testing; American Educational Research Association et al., 2014). This is important information for practitioners and test administrators in general, as it delineates the circumstances under which a nonverbal test or a translation of the test should be employed—if the participant brings certain characteristics to the test situation. Our aim in this study was therefore to investigate the relative importance of different language aspects, namely, children’s receptive language ability, expressive language ability, and bi/multilingualism,^1^ on test performance on various cognitive (i.e., intelligence, executive functions) and developmental (i.e., psychomotor skills, social-emotional skills, basic skills) functions of the Intelligence and Development Scales–2 (IDS-2; Grob & Hagmann-von Arx, 2018a). We included children’s proficiency in the test language using objective measures of receptive and expressive language abilities. Receptive language ability represents an individual’s skill in understanding others’ speech (i.e., language comprehension), while expressive language ability encompasses an individual’s skill in using speech and producing words (i.e., language production; American Psychological Association, 2015; Kauschke, 2012). The IDS-2 is a paper-and-pencil test for children and adolescents between 5 and 20 years, based on the Intelligence and Development Scales for children between 5 and 10 years (IDS; Grob et al., 2009). The series also includes a version for children between 3 and 5 years, the Intelligence and Development Scales–Preschool (IDS-P; Grob, Reimann, et al., 2013). The IDS-2 is a multidimensional psychometric tool for obtaining a comprehensive assessment of individuals’ cognitive and developmental functions using a single test battery (see Figure 1 and Table S1 in the Supplement for a summary of the IDS-2 domains included in our study; Grob & Hagmann-von Arx, 2018b). The IDS-2 was standardized between 2015 and 2017 in the German-speaking part of Switzerland, Germany, and Austria and was published in 2018. Subsequently, additional language adaptations have been released, such as in Dutch, English (UK), Italian, and Polish (Grob et al., 2018, 2019, 2021, 2022), or are currently in progress in several further countries (e.g., Brazil, Denmark, Finland, France, Norway, Sweden, Spain, and the United States).

**Figure 1.**
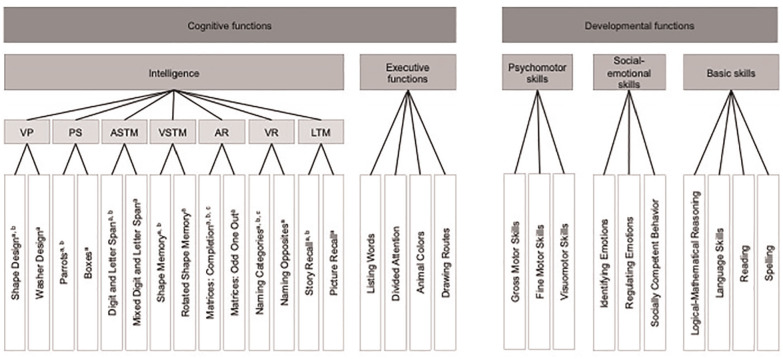
Overview of the Cognitive and Developmental Functions Assessed in the Intelligence and Development Scales–2 (IDS-2) and Included in Our Study *Note*. Functions are depicted in dark gray, domains in medium gray, intelligence group factors in light gray, and subtests in white. Reading and Spelling only for ages 7 to 20 years. The domain motivation and attitude is not shown as it is only for ages 11 to 20 years and was therefore omitted in our analyses. VP = Visual Processing; PS = Processing Speed; ASTM = Auditory Short-Term Memory; VSTM = Visuospatial Short-Term Memory; AR = Abstract Reasoning; VR = Verbal Reasoning; LTM = Long-Term Memory. ^a^Subtests included in the Profile IQ. ^b^Subtests included in the Full-Scale IQ. ^c^Subtests included in the Screening IQ.

The IDS-2 has rarely been used in previous research to investigate possible relations between language aspects and performance on the IDS-2. To date, Schweizer et al. (2021) conducted the only study so far, which examined mean-level differences between matched monolingual, simultaneously bilingual, and successively bilingual children and adolescents (each group: *n* = 132; *M*_age_ = 12.34 years) in the intelligence domain of the IDS-2. They found that successive bilinguals showed lower mean values than those of monolinguals—and to some extent also lower than those of simultaneous bilinguals—in the intelligence composites and in the group factors Verbal Reasoning and verbal Long-Term Memory. At the level of subtests, significant group differences were reported for Naming Categories, Naming Opposites, and Story Recall. No differences were detected between monolingual and simultaneously bilingual participants. However, evidence on the several other IDS-2 domains (e.g., executive functions, psychomotor skills, social-emotional skills, basic skills) is currently lacking.

With respect to the two other versions—the IDS and IDS-P—previous research examined mean-level differences between children with a migration background (defined as having a language other than German as their native language) and matched control samples (Grob, Meyer, & Hagmann-von Arx, 2013; Grob, Reimann, et al., 2013; Hagmann-von Arx et al., 2013). These studies reported no group differences in the intelligence composite (Grob, Meyer, & Hagmann-von Arx, 2013; Grob, Reimann, et al., 2013; Hagmann-von Arx et al., 2013), which assesses mainly fluid intelligence aspects (Baltes, 1987, 1990), and in most of the intelligence subtests, except for the subtest Auditory Memory. On this subtest, which measures verbal long-term memory, children with a migration background scored lower than the control group (Grob, Meyer, & Hagmann-von Arx, 2013; Grob, Reimann, et al., 2013), which has been explained by the fact that active language abilities may be necessary to solve the task (Grob, Reimann, et al., 2013). In addition, the majority of these studies found no mean-level differences in psychomotor skills, while mixed results were reported for social-emotional skills and mathematics (Grob, Meyer, & Hagmann-von Arx, 2013; Grob, Reimann, et al., 2013; Hagmann-von Arx et al., 2013).

With regard to other frequently employed intelligence tests, results are inconsistent: In the German version of the Wechsler Intelligence Scale for Children–Fifth Edition (Petermann, 2017), migration background did not explain differences between participants with and without a migration background in the intelligence composite. Instead, significant predictors were parental educational background and type of school (Daseking et al., 2018). In contrast, findings of the German version of the Wechsler Intelligence Scale for Children–Fourth Edition (Petermann & Petermann, 2011) indicated that participants with a migration background exhibited lower values in the intelligence composite than participants without a migration background (Daseking et al., 2008; Hagmann-von Arx et al., 2013).

Although previous research reported that individuals with linguistically diverse backgrounds scored lower than their monolingual peers in many domains, studies have indicated an advantage for bilingual individuals in executive functions, such as inhibitory control and cognitive flexibility (Barac et al., 2014; Grundy, 2020; Yurtsever et al., 2023). Specifically, bilingual children outperformed their monolingual peers mainly on nonverbal executive function tasks, while no differences or lower performance were found on verbal tasks (Bialystok & Craik, 2010; Foy & Mann, 2014). This advantage has been explained by the fact that both languages are constantly active in bilinguals (Kroll et al., 2012; Rodriguez-Fornells et al., 2002; Thierry & Wu, 2007), and managing these jointly activated languages results in the training of executive functions, as, for example, responding in the target language has to be controlled (Abutalebi & Green, 2008).

Concerning basic skills, language abilities are crucial for learning processes in school and hence for the successful acquisition of reading, spelling, and mathematics (e.g., Storch & Whitehurst, 2002). Previous research indicated that multilingual individuals and individuals with a migration background achieved lower test scores than their control peers in reading and spelling (Konsortium PISA.ch, 2019; Melby-Lervåg & Lervåg, 2014; Verhoeven, 2000). Moreover, there is evidence that vocabulary size relates to scholastic performance (e.g., Kastner et al., 2001) and the acquirement of literacy skills (Lee Swanson et al., 2008; Ouellette, 2006). Although previous studies on the IDS and IDS-P produced inconsistent results with respect to mathematics (Grob, Meyer, & Hagmann-von Arx, 2013; Grob, Reimann, et al., 2013; Hagmann-von Arx et al., 2013), several other studies reported group differences between individuals with and without a migration background in the natural sciences (Konsortium PISA.ch, 2018) and mathematics (e.g., Bos et al., 2007; Ehm et al., 2011; Paetsch et al., 2016). Moreover, basic skills are also described as “cultural skills,” which seem to be related to previous language experiences in the school context (Grob & Hagmann-von Arx, 2018b; Köller & Baumert, 2008).

Nevertheless, a large part of the existing literature usually focused on group differences, such as between individuals with and without a migration background (Grob, Meyer, & Hagmann-von Arx, 2013; Grob, Reimann, et al., 2013; Hagmann-von Arx et al., 2013) or between monolinguals and bilinguals (Schweizer et al., 2021). This makes it difficult to disentangle the relative importance of language aspects in test performance. Moreover, individuals within these groups may also have distinct levels of proficiency in the test language and therefore be at different positions on the continuum of language abilities (Ortiz, 2019). This has not been taken into account in analyses comparing groups. However, to derive concrete recommendations for practice, a closer look at individuals’ measurable language abilities has been suggested (Ortiz, 2019). To the best of our knowledge, only one recent study (Cormier et al., 2022) followed this approach and investigated the effects of participants’ receptive and expressive language abilities in English on test performance in the Woodcock–Johnson Tests of Cognitive Abilities (4th ed.; Schrank et al., 2014). Results of this study indicated that both language abilities were significantly related to test performance in this cognitive test battery, with receptive language ability showing slightly higher associations than expressive language ability. However, the authors noted in their limitations section that the sample did not include many English-language learners (Cormier et al., 2022), which might have restricted the generalizability of the findings. Moreover, these results also raise the question to what extent objective measures of receptive and expressive language abilities relate to performance in other important domains of the child development, beyond cognitive abilities.

## The Present Study

Building on this literature, we aimed to examine how different language aspects (i.e., children’s receptive language ability, expressive language ability, and bi/multilingualism) contribute beyond each other to test performance on the cognitive and developmental functions assessed with the IDS-2 (Grob & Hagmann-von Arx, 2018a). We thereby sought to follow a holistic approach by integrating multiple language aspects on one side and various cognitive and developmental domains on the other. Specifically, we investigated the relations of distinct language abilities to test scores on the IDS-2 after controlling for relevant sociodemographic variables (i.e., sex and socioeconomic status [SES], represented by maternal educational background; Weiss & Saklofske, 2020).^2^ We distinguished between children’s receptive and expressive language abilities and relied on standardized assessments of these two language skills. Moreover, we added bi/multilingualism in the final step to explore whether other components of having a linguistically diverse background contribute beyond objectively measured language abilities to the variance in test scores on the IDS-2. We employed the IDS-2 because this test battery offers a comprehensive assessment of important cognitive and developmental functions (see Figure 1 for an overview; Grob & Hagmann-von Arx, 2018b). In addition, this test procedure is widely used by clinicians, such as psychologists and physicians, in countries that have a high number of immigrants (i.e., Switzerland, Germany, and Austria; Austrian Federal Statistical Office, 2022; German Federal Statistical Office, 2023; Swiss Federal Statistical Office, 2022a) and therefore many children with linguistically diverse backgrounds in psychological assessment settings. Hence, it is crucial that clinicians are aware of the composites, group factors, and subtests that relate to aspects of language to take these associations into account when administering the IDS-2 to participants at risk for linguistic disadvantages. To uncover potential differential effects on the composites, group factors, and subtests of the IDS-2, we adopted a fine-grained level of analysis that includes all possible scores, as results may vary across these different levels and scores. Specifically, with respect to the three intelligence composites Profile IQ, Full-Scale IQ, and Screening IQ, they are based on distinct sets of subtests with different content and input. Research has demonstrated that internal score comparability of intelligence composites may be constrained at the individual-level (Grieder et al., 2022), implying that these scores are not interchangeable and should be analyzed individually. However, there is currently no evidence concerning the relative importance of language abilities and bi/multilingualism on children’s test performance in the IDS-2 intelligence domain, and relations between language aspects and the other IDS-2 domains have not yet been investigated. Thus, we also aimed to extend current knowledge regarding the validity of test-score interpretations of the IDS-2.

In line with previous research (e.g., Hagmann-von Arx et al., 2013), we assumed that as the verbal demands of a task increase—with respect to instructions, content, and response format—language aspects will be more strongly associated with the test score and explain more variance. Therefore, we hypothesized that performance on those subtests and intelligence group factors of the IDS-2 that we classified as “low linguistic loading” (see Table 1) would show the smallest positive associations with language aspects. In contrast, we expected that performance on subtests and intelligence group factors of the IDS-2 that we categorized as “high linguistic loading” (see Table 1) would show the strongest positive associations with language aspects. We based our classification on findings from previous studies presented above and on considerations of the verbal demands of the specific tasks.^3^ To explore expressive language ability and bi/multilingualism as incremental predictors, we formulated two research questions: Does expressive language ability explain additional variance in test scores on the IDS-2 beyond receptive language ability (Research Question 1)? Does bi/multilingualism explain additional variance in test scores on the IDS-2 beyond receptive and expressive language abilities (Research Question 2)?

**Table 1:** Cognitive and Developmental Functions From the Intelligence and Development Scales–2 Ranked According to Their Assumed Degree of Linguistic Loading

Domain	Low linguistic loading variable	Moderate linguistic loading variable	High linguistic loading variable
Intelligence	*Visual Processing*	*Auditory Short-Term Memory*	*Verbal Reasoning*
	Shape Design	Digit and Letter Span	Naming Categories
	Washer Design	Mixed Digit and Letter Span	Naming Opposites
	*Processing Speed*	*Long-Term Memory*	*Long-Term Memory*
	Parrots	Picture Recall	Story Recall
	Boxes		
	*Abstract Reasoning*		
	Matrices: Completion		
	Matrices: Odd One Out		
	*Visuospatial Short-Term Memory*		
	Shape Memory		
	Rotated Shape Memory		
Executive functions	Animal Colors	Listing Words	
	Drawing Routes	Divided Attention	
Psychomotor skills	Gross Motor Skills		
	Fine Motor Skills		
	Visuomotor Skills		
Social-emotional skills		Identifying Emotions	
		Regulating Emotions	
		Socially Competent Behavior	
Basic skills			Logical-Mathematical Reasoning
		
			Reading
			Spelling

*Note.* It is assumed that performances in subtests and in intelligence group factors of the IDS-2 that are classified as “low linguistic loading” would be least susceptible to language effects, whereas performances in subtests and in intelligence group factors of the IDS-2 that are classified as “high linguistic loading” would be most susceptible to language effects. Long-Term Memory is listed in two categories as it contains subtests with different linguistic loadings. Intelligence group factors are printed in italics.

## Method

We report how we determined our sample size, all data exclusions, all manipulations, and all measures in the study.

### Participants

The sample consisted of participants from the IDS-2 standardization and validation study (*N* = 2,030, 52% female, age range 5–20 years). As the IDS-2 subtest Language Skills is administered only to participants aged 5–10 years, we omitted adolescents aged 11–20 years (*n* = 1,120) from our analyses. In addition, we excluded participants with missing data concerning their receptive and expressive language abilities, native language, age, sex, or maternal educational background (*n* = 84). The final sample comprised 826 children (*M*_age_ = 8.06 years, *SD* = 1.66, age range: 5.02–10.99 years) with 51% being female. In terms of education, 59.6% of the participants’ mothers had not gone beyond mandatory education or had vocational job training (i.e., no postsecondary education), while 40.4% of the participants’ mothers had obtained a university degree (i.e., postsecondary education), which is higher than in the corresponding population (e.g., 29.2% of women currently hold a postsecondary degree in Switzerland; Swiss Federal Statistical Office, 2022b). Regarding children’s current education, they attended preschool (*n* = 5), kindergarten (*n* = 189), elementary school (*n* = 590), secondary school (*n* = 14), special education services (*n* = 23), other (*n* = 3), or unknown (*n* = 2). About 15% of the sample reported one or more medical or psychological diagnosis.^4^

Participants were from the German-speaking part of Switzerland (*n* = 529), Germany (*n* = 252), and Austria (*n* = 45). Of the children, 48.8% held Swiss nationality, 33.1% were of German nationality, and 18.1% indicated having a different nationality. A total of 26.0% reported being multilingual (bilingual: 24.4%; trilingual or more than three languages: 1.6%), which we defined as speaking German (the test language) and having at least one other language as their native language (hereafter denoted: bi/multilingual children). This percentage corresponds to recent studies on the proportion of bi/multilingual individuals in the corresponding population (e.g., in Switzerland, 33% of children under the age of 15 years were exposed to two languages at home; in Germany, 21% of children under the age of 14 years spoke a language other than German in the family context; German Federal Statistical Office, 2022; Swiss Federal Statistical Office, 2021). Besides German, the children in our study spoke Russian (*n* = 32), English (*n* = 29), French (*n* = 26), Italian (*n* = 19), Turkish (*n* = 18), Spanish (*n* = 14), Albanian (*n* = 12), Serbian (*n* = 12), Tamil (*n* = 9), Portuguese (*n* = 7), Dutch (*n* = 5), Chinese (*n* = 4), Croatian (*n* = 4), and 23 other languages (each under *n* = 4). However, all participants had to speak the test language (i.e., German) sufficiently to take part in the study. Hence, all participants’ educational language was German. The other 74.0% reported being monolingual German speakers and indicated German as their native language. Characteristics of monolingual and bi/multilingual children are presented in Table S2 in the Supplement.

### Procedure

Recruitment was conducted between 2015 and 2017 through day care centers, playgroups, kindergartens, schools, and school psychological services in Switzerland as well as through psychosocial institutions and universities in Germany and Austria. Participants were individually tested with the IDS-2 at their homes, at a laboratory of the university, or at the respective institutions’ laboratories by one of several trained test administrators (i.e., undergraduate psychology students or psychologists). The administration of the IDS-2 took about 4 h, depending on participants’ age and performance, as there are age-specific implementation rules and performance-based rules for ending testing. Testing could be divided into two sessions one week apart if the participant wished. Parents were asked to report demographic variables, such as their children’s native language(s) and parental educational background, in a questionnaire administered by the test administrator prior to testing. The families received a monetary incentive for participation either as a gift card or in cash. The local ethics committee (Ethics Committee Northwest and Central Switzerland) approved the study protocol. Parents were asked to sign a consent form.

### Instrument

The IDS-2 (Grob & Hagmann-von Arx, 2018a) assesses cognitive (i.e., intelligence and executive functions) and developmental (i.e., psychomotor skills, social-emotional skills, basic skills, and motivation and attitude) functions in children and adolescents aged 5 to 20 years. An overview and a detailed description of the IDS-2 domains included in our study are provided in Figure 1 and Supplemental Table S1, respectively. The IDS-2 also assesses participants’ language abilities in terms of their level of receptive language ability and expressive language ability. Children’s receptive language ability is measured by asking them to carry out recited instructions using multiple materials. First, participants name the items presented. Then, they are asked to play with the items according to the instructions given by the test administrator, as for example: “The ball is rolling around the pencil”. Participants can start playing when the sentence is finished. Sentences must not be repeated. To assess children’s expressive language ability, children are asked to form meaningful sentences from spoken and pictorially depicted words. For example, participants see a picture of a woman and a key, while the test administrator says: “Now say something that makes sense and in which a woman and a key appear together”. Grammatical errors, omission of given words, simple listing of words, meaningless sentences, and incoherent sentences are rated as errors.

Several studies have documented the psychometric properties of the IDS-2 for the standardization sample (e.g., Grieder & Grob, 2020; Odermatt et al., 2022), showing, for example, high correlations with other frequently used test procedures in German-speaking countries (Grob & Hagmann-von Arx, 2018b).

### Statistical Analyses

Hierarchical regression analyses were conducted to examine whether different language variables (i.e., receptive language ability, expressive language ability, and bi/multilingualism) were associated with scores on composites, intelligence group factors, and subtests of the cognitive and developmental functions of the IDS-2, beyond sex and SES. We used age-standardized IDS-2 scores (*M* = 100, *SD* = 15, for Profile IQ, Full-Scale IQ, Screening IQ, and the seven intelligence group factors; *M* = 10, *SD* = 3, for other composites and subtests). We formed the variable *bi/multilingualism* using information regarding children’s native language reported by the parents. In all regression analyses, we entered control variables (i.e., sex and SES) in Step 1, children’s receptive language ability in Step 2, and their expressive language ability in Step 3. Bi/multilingualism (i.e., being bi/multilingual vs. being monolingual) was added in Step 4.^5^ We calculated separate analyses for each composite, intelligence group factor, and subtest of the IDS-2, resulting in 40 analyses.^6^ To adjust for multiple testing, we corrected *p* values (hereafter denoted *p*_H_) according to Hommel (1988). Moreover, reliabilities for the IDS-2 scores were indicated, including Cronbach’s alpha for homogeneous subtests, reliabilities computed using a formula by Lienert and Raatz (1998) for composites, and retest reliabilities for single-item or heterogeneous subtests obtained from the IDS-2 manual (Grob & Hagmann-von Arx, 2018b). Analyses were performed using *R* (R Core Team, 2022).

## Results

**Figure 2.**
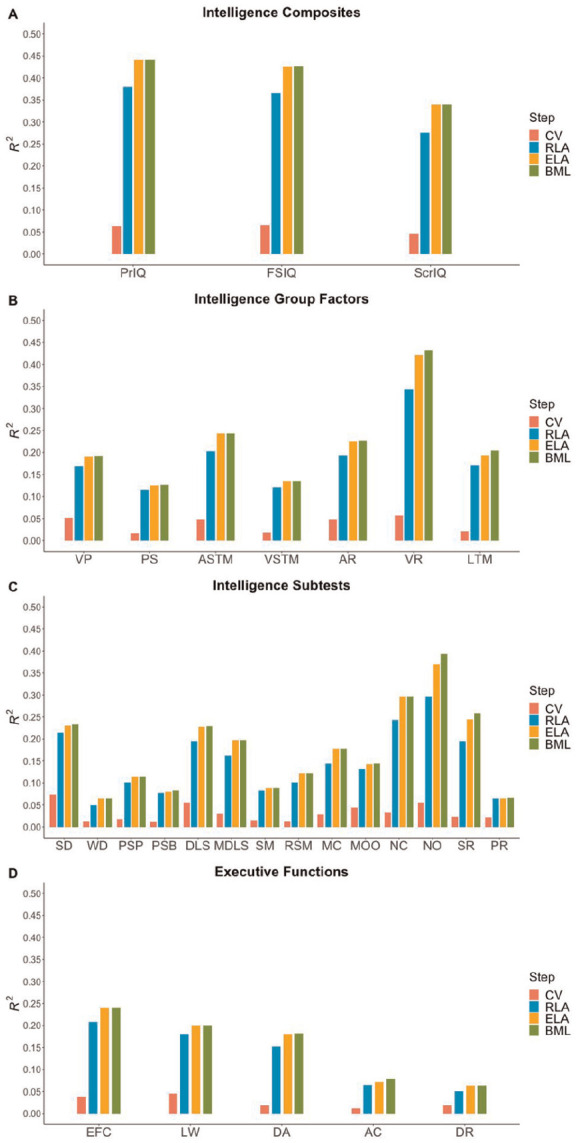
The Proportion of Cumulative Variance Explained by Each Predictor in the Scores on the Cognitive Functions From the Intelligence and Development Scales–2 *Note*. The cumulative explained variance by each of the four predictors (i.e., Step 1: control variables, Step 2: receptive language ability, Step 3: expressive language ability, Step 4: bi/multilingualism) is shown for (A) intelligence composites, (B) intelligence group factors, (C) intelligence subtests, and (D) executive functions composite and subtests of the Intelligence and Development Scales–2. CV = control variables (i.e., sex and socioeconomic status); RLA = receptive language ability; ELA = expressive language ability; BML = bi/multilingualism; PrIQ = Profile IQ; FSIQ = Full-Scale IQ; ScrIQ = Screening IQ; VP = Visual Processing; PS = Processing Speed; ASTM = Auditory Short-Term Memory; VSTM = Visuospatial Short-Term Memory; AR = Abstract Reasoning; VR = Verbal Reasoning; LTM = Long-Term Memory; *SD* = Shape Design; WD = Washer Design; PSP = Parrots; PSB = Boxes; DLS = Digit and Letter Span; MDLS = Mixed Digit and Letter Span; SM = Shape Memory; RSM = Rotated Shape Memory; MC = Matrices: Completion; MOO = Matrices: Odd One Out; NC = Naming Categories; NO = Naming Opposites; SR = Story Recall; PR = Picture Recall; EFC = Executive functions composite; LW = Listing Words; DA = Divided Attention; AC = Animal Colors; DR = Drawing Routes.

**Figure 3.**
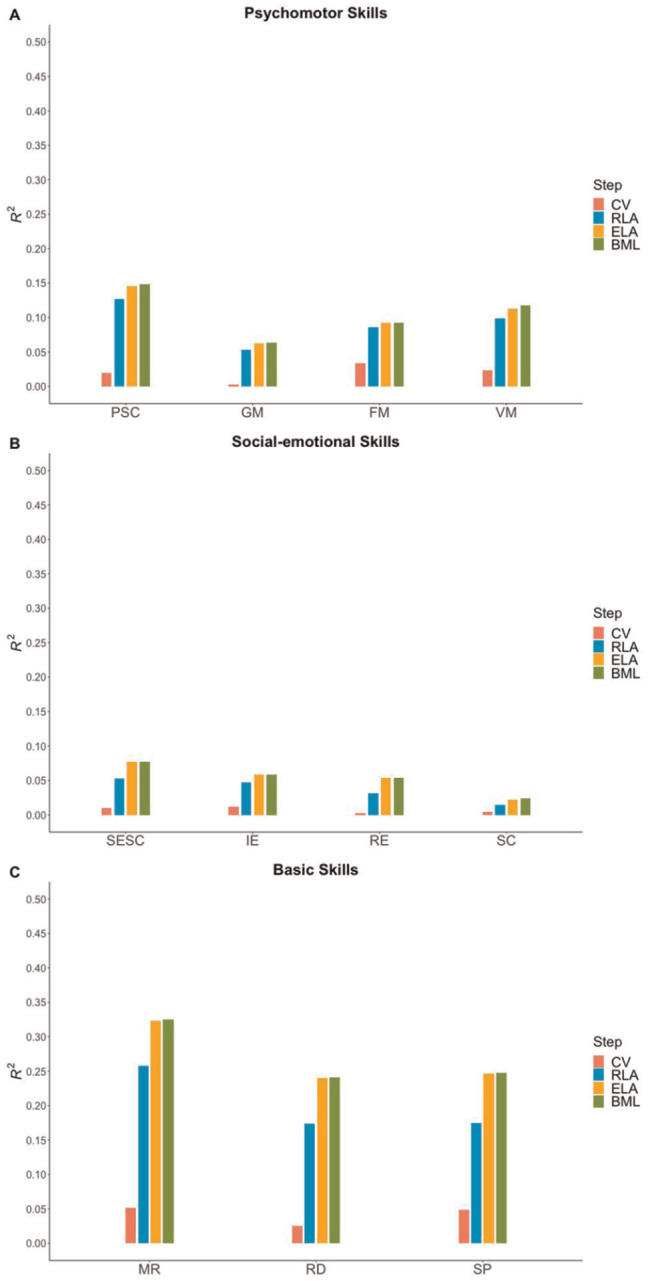
The Proportion of Cumulative Variance Explained by Each Predictor in the Scores on the Developmental Functions From the Intelligence and Development Scales–2 *Note*. The cumulative explained variance by each of the four predictors (i.e., Step 1: control variables, Step 2: receptive language ability, Step 3: expressive language ability, Step 4: bi/multilingualism) is shown for (A) psychomotor skills composite and subtests, (B) social-emotional skills composite and subtests, and (C) basic skills subtests of the Intelligence and Development Scales–2. CV = control variables (i.e., sex and socioeconomic status); RLA = receptive language ability; ELA = expressive language ability; BML = bi/multilingualism; PSC = Psychomotor skills composite; GM = Gross Motor Skills; FM = Fine Motor Skills; VM = Visuomotor Skills; SESC = Social-emotional skills composite; IE = Identifying Emotions; RE = Regulating Emotions; SC = Socially Competent Behavior; MR = Logical-Mathematical Reasoning; RD = Reading; SP = Spelling.

Table 2 presents the descriptive statistics of the predictors (i.e., sex, SES, receptive and expressive language abilities, bi/multilingualism) and the IDS-2 scores. Reliability coefficients were high for composites and intelligence group factors and high to satisfactory for subtests of the IDS-2. Pearson correlations showed moderate associations between receptive language ability, expressive language ability, and bi/multilingualism and therefore no multicollinearity between the language aspects was detected (see Table 3).^7^ The proportion of cumulative variance explained (*R*^2^) by each predictor in the IDS-2 scores is displayed in Figure 2 for the cognitive functions and in Figure 3 for the developmental functions. Results of the hierarchical regression analyses are reported in Tables S8 to S15 in the Supplement.^8^

**Table 2:** Descriptive Statistics of Predictors and Composites, Intelligence Group Factors, and Subtests From the Intelligence and Development Scales–2 (IDS-2)

Predictor or score	*M*	*SD*	*n*	%	Minimum	Maximum	Skewness	Kurtosis	Reliability
Predictor									
Sex (female)			424	51.3%					
SES (no postsecondary maternal education)			492	59.6%					
Receptive language ability^a^	10.33	2.99			1	19	–0.25	0.43	0.88
Expressive language ability^a^	10.28	3.08			1	19	–0.46	0.06	0.92
Bi/multilingualism (yes)			215	26.0%					
IDS-2 scores									
Profile IQ^b^	100.42	15.28			55	145	–0.55	0.52	0.99
Full-Scale IQ^b^	100.41	15.3			55	142	–0.47	0.27	0.98
Screening IQ^b^	100.58	15.25			55	145	–0.19	0.12	0.97
Visual Processing^b^	99.75	15.67			55	141	–0.19	0.16	0.97
Processing Speed^b^	100.06	15.3			55	145	–0.1	0.53	0.97
Auditory Short-Term Memory^b^	100.53	15.25			55	145	–0.15	0.01	0.96
Visuospatial Short-Term Memory^b^	100.07	14.65			55	145	–0.14	0.57	0.95
Abstract Reasoning^b^	100.06	15.67			55	145	–0.04	0.19	0.97
Verbal Reasoning^b^	100.46	14.4			55	139	–0.54	0.46	0.97
Long-Term memory^b^	100.03	14.74			55	142	–0.23	0.29	0.97
Shape Design^a^	10.18	3.15			1	19	–0.18	0.34	0.88
Washer Design^a^	10.29	3.08			1	19	–0.06	0.16	0.88
Parrots^a^	10.20	3.00			1	19	–0.34	0.57	0.87
Boxes^a^	10.09	3.07			1	19	–0.16	0.89	0.86
Digit and Letter Span^a^	10.50	2.84			1	19	–0.14	0.15	0.83
Mixed Digit and Letter Span^a^	10.66	2.94			1	19	–0.24	0.07	0.80
Shape Memory^a^	10.33	2.91			1	19	–0.01	0.69	0.80
Rotated Shape Memory^a^	10.22	2.41			1	19	–0.25	0.92	0.80
Matrices: Completion^a^	10.50	3.12			1	19	0.17	0.18	0.88
Matrices: Odd One Out^a^	10.26	3.01			1	19	0.09	0.24	0.88
Naming Categories^a^	10.51	2.89			1	19	–0.41	0.64	0.89
Naming Opposites^a^	10.56	2.91			1	17	–0.75	0.93	0.85
Story Recall^a^	10.23	3.04			1	19	–0.38	0.47	0.92
Picture Recall^a^	10.52	2.89			1	19	0.04	0.34	0.88
Executive functions composite^b^	10.11	1.90			3.12	15.75	–0.28	0.35	0.97
Listing Words^c^	9.93	3.07			1	18	–0.09	–0.14	0.75
Divided Attention^b^	10.11	2.31			1.5	18	–0.45	0.96	0.91
Animal Colors^c^	10.08	3.03			1	19	0.01	0.04	0.72
Drawing Routes^b^	10.34	2.49			2.5	17	–0.33	–0.11	0.95
Psychomotor skills composite^b^	10.33	1.86			2.67	16.83	–0.32	0.6	0.97
Gross Motor Skills^a^	10.51	3.12			1	19	–0.1	0.03	0.71
Fine Motor Skills^b^	10.35	2.39			1.5	19	–0.1	0.4	0.95
Visuomotor Skills^b^	10.14	2.08			2.5	19	–0.11	0.69	0.94
Social-emotional skills composite^b^	10.50	1.92			1.33	14.67	–1.15	2.55	0.95
Identifying Emotions^c^	10.29	2.37			1	14	–1.43	2.19	0.85
Regulating Emotions^c^	10.49	2.66			1	15	–0.89	0.71	0.78
Socially Competent Behavior^c^	10.73	2.9			1	19	–0.58	1.06	0.71
Basic skills composite^b^	10.13	2.33			1	17	–0.42	0.88	0.98
Logical-Mathematical Reasoning^a^	10.22	3.03			1	19	–0.1	0.37	0.94
Reading^b^	9.72	2.73			1	17.5	–0.36	0.33	0.97
Spelling^a^	10.15	3.11			1	19	–0.38	0.49	0.72

*Note.* SES = socioeconomic status.

a Cronbach’s alpha reported.

b Reported reliability calculated according to a formula by Lienert and Raatz (1998).

c Retest reliability reported.

**Table 3:** Pearson Correlations of the Predictors Included in the Hierarchical Regression Analyses

Variable		1	2	3	4	5	6
1	Sex	—					
2	SES	.03	—				
3	RLA	.12***	.16***	—			
4	ELA	.11**	.17***	.45***	—		
5	BML	.05	–.04	.20***	.23***	—	
6	IQ	.05	.25***	.60***	.50***	.16***	
7	Age	–.02	–.03	–.01	–.01	.03	–.02

*Note.* Sex: -1 = male, 1 = female; SES = socioeconomic status: –1 = no postsecondary maternal education, 1 = postsecondary maternal education; BML = bi/multilingualism: –1 = yes, 1 = no. RLA = receptive language ability; ELA = expressive language ability; IQ = intelligence (Profile IQ).

** *p* < .01. ***p < .001.

### Cognitive Functions

#### Intelligence

After controlling for sex and SES, children’s receptive language ability was significantly associated with higher scores on Profile IQ (β = .57, *p*_H_ < .001), Full-Scale IQ (β = .56, *p*_H_ < .001), and Screening IQ (β = .49, *p*_H_ < .001), explaining between 23% and 32% of additional variance. Children’s receptive language ability was also significantly related to each intelligence group factor score (highest for Verbal Reasoning: β = .55, *p*_H_ < .001; lowest for Processing Speed: β = .32, *p*_H_ < .001) and each subtest score (highest for Naming Opposites: β = .50, *p*_H_ < .001; lowest for Washer Design: β = .20, *p*_H_ < .001). Additional explained variance ranged between 4% and 28% beyond control variables. Expressive language ability, entered in Step 3, significantly correlated with higher scores on Profile IQ (β = .28, *p*_H_ < .001), Full-Scale IQ (β = .28, *p*_H_ < .001), and Screening IQ (β = .28, *p*_H_ < .001), accounting for 6% of additional variance in each composite beyond control variables and receptive language ability. Expressive language ability was related to scores on each intelligence group factor, except for Processing Speed (β = .11, *p*_H_ = .665) and Visuospatial Short-Term Memory (β = .13, *p*_H_ = .111), additionally explaining between 2% and 8% of variance. At the level of subtests, children’s expressive language ability was associated with scores on Shape Design (β = .14, *p*_H_ = .024), Digit and Letter Span (β = .20, *p*_H_ < .001), Matrices: Completion (β = .21, *p*_H_ < .001), Naming Categories (β = .26, *p*_H_ < .001), Story Recall (β = .25, *p*_H_ < .001), Mixed Digit and Letter Span (β = .21, *p*_H_ < .001), Rotated Shape Memory (β = .16, *p*_H_ = .006), and Naming Opposites (β = .31, *p*_H_ < .001). Between 2% and 7% of variance was explained beyond control variables and children’s receptive language ability. Adding the variable bi/multilingualism in Step 4 only explained variance in the subtests Story Recall (β = .13, *p*_H_ = .022) and Naming Opposites (β = .16, *p*_H_ < .001), accounting for 2% of additional variance in each of these subtests beyond control variables and receptive and expressive language abilities. No significant associations were found between bi/multilingualism and scores on the intelligence composites or group factors in Step 4 (see Figure 2 and Tables S8 to S11 in the Supplement).

#### Executive Functions

Beyond the control variables sex and SES, children’s receptive language ability was significantly related to higher scores on the executive functions composite (β = .42, *p*_H_ < .001) and on each subtest in the executive functions domain (highest for Listing Words: β = .38, *p*_H_ < .001; lowest for Drawing Routes: β = .18, *p*_H_ < .001), explaining between 3% and 17% of additional variance. In Step 3, children’s expressive language ability was associated with scores on the executive functions composite (β = .20, *p*_H_ < .001) as well as with scores on the subtests Listing Words (β = .16, *p*_H_ = .007) and Divided Attention (β = .19, *p*_H_ < .001), explaining between 2% and 3% of additional variance beyond control variables and children’s receptive language ability. The variable bi/multilingualism did not account for additional variance in any of the scores in the executive functions domain in Step 4 beyond control variables and receptive and expressive language abilities (see Figure 2 and Table S12 in the Supplement).

### Developmental Functions

#### Psychomotor Skills

Concerning psychomotor skills, positive associations between children’s receptive language ability and scores on the psychomotor skills composite (β = .33, *p*_H_ < .001) as well as all subtests (highest for Visuomotor Skills: β = .28, *p*_H_ < .001) were found, explaining between 5% and 11% of additional variance beyond the control variables. Expressive language ability, entered in Step 3, was related only to scores on the psychomotor skills composite (β = .15, *p*_H_ = .010; Δ*R*^2^ *=* 2%) and not to scores on the subtests beyond control variables and children’s receptive language ability. The variable bi/multilingualism did not explain any additional variance in the scores in the psychomotor skills domain in Step 4 beyond control variables and receptive and expressive language abilities (see Figure 3 and Table S13 in the Supplement).

#### Social-Emotional Skills

After controlling for sex and SES, children’s receptive language ability was significantly associated with scores on the social-emotional skills composite (β = .21, *p*_H_ < .001) and the subtests Identifying Emotions (β = .19, *p*_H_ < .001) and Regulating Emotions (β = .17, *p*_H_ < .001), explaining between 3% and 4% of additional variance. No relationship was found with scores on the subtest Socially Competent Behavior (β = .10, *p*_H_ = .742). When children’s expressive language ability was entered in Step 3, significant correlations were detected only with scores on the social-emotional skills composite (β = .18, *p*_H_ = .002) and the subtest Regulating Emotions (β = .17, *p*_H_ = .006), explaining between 2% and 3% of additional variance beyond control variables and children’s receptive language ability. The variable bi/multilingualism did not explain additional variance in any of the scores in the social-emotional skills domain in Step 4 beyond control variables and receptive and expressive language abilities (see Figure 3 and Table S14 in the Supplement).

#### Basic Skills

Regarding basic skills, children’s receptive language ability was significantly related to higher scores on each subtest (highest for Logical-Mathematical Reasoning: β = .46, *p*_H_ < .001), explaining between 12% and 21% of additional variance beyond control variables. In Step 3, expressive language ability was also significantly correlated with higher scores on each subtest (highest for Spelling: β = .31, *p_H_* < .001) and accounted for between 6% and 8% of additional variance beyond control variables and children’s receptive language ability. No significant associations were found when the variable bi/multilingualism was entered in Step 4 after accounting for control variables and receptive and expressive language abilities (see Figure 3 and Table S15 in the Supplement).

## Discussion

In this study, we sought to investigate the relative importance of different language aspects (i.e., receptive and expressive language abilities, bi/multilingualism) for participants’ test performance on various cognitive and developmental functions of the IDS-2 using a sample of children aged 5 to 10 years. Hierarchical regression analyses showed that children’s receptive language ability was significantly related to all scores on the IDS-2 when accounting for sex and SES, except for the subtest Socially Competent Behavior. Children’s expressive language ability explained overall little additional variance above control variables and receptive language ability in the IDS-2 scores. The highest amounts of additional explained variance were found for intelligence composites, the intelligence group factor Verbal Reasoning and its corresponding subtests Naming Categories and Naming Opposites, and the subtests in the basic skills domain (i.e., Logical-Mathematical Reasoning, Reading, and Spelling). In contrast, bi/multilingualism, entered in the final step, explained variance beyond control variables and receptive and expressive language abilities solely in the two intelligence subtests Story Recall and Naming Opposites. Overall, language aspects explained the largest variance in scores in the intelligence and basic skills domains. In addition, the magnitude of our findings (2%–32% explained variance) was comparable to that of a similar study (Cormier et al., 2022), in which the inclusion of receptive and expressive language abilities explained between 4% and 40% of additional variance on test performance in the Woodcock–Johnson Tests of Cognitive Abilities (4th ed.; Schrank et al., 2014). The results emphasize the relevance of considering children’s proficiency in the test language within the assessment of cognitive and developmental functions with the IDS-2, especially in tasks with high verbal demands, when testing participants at risk for linguistic disadvantages.

### Receptive Language Ability and Test Performance on the IDS-2

In line with previous research (Cormier et al., 2022), children’s receptive language ability explained variance in scores on almost all subtests, intelligence group factors, and composites of the IDS-2 beyond sex and SES. Hence, the ability to understand verbal instructions is crucial to completing the IDS-2 tasks and therefore for children’s test performance. This can be explained by the fact that all test directions are given verbally in the IDS-2. Moreover, about one third and approximately a quarter of the variance in scores in the intelligence and basic skills domains, respectively, was explained by receptive language ability. With respect to the intelligence domain, especially in the intelligence composites Profile IQ, Full-Scale IQ, and Screening IQ as well as in the group factor Verbal Reasoning and its corresponding subtests Naming Categories and Naming Opposites, high associations were found with children’s receptive language ability. These findings support our hypotheses that Verbal Reasoning and its subtests show high linguistic loadings (see Table 1) and are in line with previous research on group differences in the IDS-2 intelligence domain by Schweizer and colleagues (2021). As the intelligence group factor Verbal Reasoning is designed to measure the broad ability Comprehension-Knowledge according to the Cattell–Horn–Carroll (CHC) model (McGrew, 1997, 2009; Schneider & McGrew, 2018), which encompasses language-based knowledge (Schneider & McGrew, 2018), mainly crystallized and verbal aspects of intelligence are covered. In comparison to Verbal Reasoning and its subtests, a slightly lower amount of explained variance was detected for Story Recall. However, this variable was also classified as “high linguistic loading variable” (see Table 1), and therefore, our hypothesis for Story Recall is only partially fulfilled. Nevertheless, bi/multilingualism explained variance beyond control variables and language abilities in this subtest. This suggests that other components of having a linguistically diverse background may be more relevant.

In terms of basic skills, children’s receptive language ability explained variance in scores on all subtests, including Logical-Mathematical Reasoning, Reading, and Spelling, beyond control variables. These findings are in line with previous research that has emphasized the importance of language abilities for the acquirement of basic skills (e.g., Storch & Whitehurst, 2002). Moreover, they are consistent with our hypotheses (see Table 1) where we classified the basic skills subtests as “high linguistic loading variables”. Nevertheless, compared to Verbal Reasoning and its subtests, we found lower amounts of explained variance for both Reading and Spelling, while the amount of explained variance was comparable for Logical-Mathematical Reasoning. In the case of Logical-Mathematical Reasoning, we found one of the highest associations with children’s receptive language ability across all analyses. One reason for our finding might be that the items in this subtest are presented according to a flexible interview approach (Ginsburg, 1997). This includes test instructions with extensive verbal explanations and the possibility of asking children specific questions about the solution paths they used. Therefore, a high level of language comprehension may be required to understand the tasks of the Logical-Mathematical Reasoning subtest in the IDS-2. However, as previous research on the IDS and IDS-P has provided mixed evidence (Grob, Meyer, & Hagmann-von Arx, 2013; Grob, Reimann, et al., 2013; Hagmann-von Arx et al., 2013), future studies are needed to clarify these inconsistencies.

In addition, children’s receptive language ability was related to all scores in the executive functions domain, with the highest amounts of variance explained in the composite and in the subtests Listing Words and Divided Attention beyond sex and SES which was in line with our hypotheses (see Table 1). As both of these subtests include long and to some degree complex instructions, more language comprehension is demanded from the participant. Moreover, one explanation for the smaller associations between receptive language ability and scores on the other executive function subtests, Animal Colors and Drawing Routes, may be that these include pictorially depicted tasks, which might help participants understand test directions.

In accordance with our hypotheses (see Table 1), children’s receptive language ability explained comparatively little additional variance in scores on subtests in the psychomotor skills domain. This result corresponds to studies that reported no mean-level differences between children with a migration background and control samples in the IDS and IDS-P psychomotor skills domains (Grob, Reimann, et al., 2013; Hagmann-von Arx et al., 2013). One explanation for this finding might be that in tasks in the IDS-2 psychomotor skills domain, test directions are accompanied by gestures from the test administrator, which serve to demonstrate the task during the instruction phase (e.g., test administrators show how to balance on a rope before the testing starts).

Concerning the social-emotional skills domain, children’s receptive language ability explained the lowest amounts of variance beyond control variables in scores in this domain. This is in contrast to our hypotheses where we classified social-emotional skills subtests as “moderate linguistic loading variables” (see Table 1). Hence, children’s language comprehension skills play a minor role in performance on these IDS-2 tasks. For the Socially Competent Behavior subtest, we did not find a significant association with children’s receptive language ability, representing the only nonsignificant association with respect to this variable. One reason for this finding may be that the instructions in this subtest are presented with detailed pictorial illustrations of social situations, which might have helped participants understand the verbal directions. Moreover, the results for the social-emotional skills domain of the IDS reported in previous studies are inconsistent (Grob, Meyer, & Hagmann-von Arx, 2013; Hagmann-von Arx et al., 2013). For example, one study found a significant mean-level difference between participants with and without a migration background in the composite score in the IDS social emotional skills domain in older children aged 9 to 10 years, but not in younger children between 6 and 8 years (Hagmann-von Arx et al., 2013). However, they did not examine group differences at the subtest level. Since the few previous studies have provided mixed evidence, this finding should be treated with caution and ought to be replicated by further studies.

### Expressive Language Ability and Test Performance on the IDS-2

Children’s expressive language ability explained only little additional variance beyond sex, SES, and children’s receptive language ability in most of the IDS-2 scores (Research Question 1). Nevertheless, in accordance with previous research (e.g., Melby-Lervåg & Lervåg, 2014; Paetsch et al., 2016; Schweizer et al., 2021; Verhoeven, 2000), we observed some considerable levels of additional explained variance in the intelligence group factor Verbal Reasoning and its corresponding subtests Naming Categories and Naming Opposites as well as in the basic skills subtests Logical-Mathematical Reasoning, Reading, and Spelling. Thus, these tasks require not only the ability to understand but also the ability to produce verbal information.

For example, concerning basic skills, children have to verbally explain their solution processes in the subtest Logical-Mathematical Reasoning, or they need to answer questions about a previously read text in the subtest Reading. The strong associations we have found between receptive and expressive language abilities and basic skills might also be explained by the fact that language abilities are generally central to learning in the school context, not only for understanding the content of lessons but also for being able to communicate with others about it (Paetsch et al., 2016). Concerning the subtests Naming Categories and Naming Opposites, the participant has to answer the questions verbally, with no opportunity to respond nonverbally. In addition, the quality of the verbal response is also evaluated and embedded as part of the task content, as vocabulary and verbal logical reasoning are required (Grob & Hagmann-von Arx, 2018b). Hence, the linguistic demands represent an element of the measurement *and* an aspect of the construct itself that is intended to be assessed in these two subtests.

### Bi/Multilingualism and Test Performance on the IDS-2

Finally, bi/multilingualism explained variance beyond control variables and receptive and expressive language abilities in only two subtests of the intelligence domain, namely, Story Recall and Naming Opposites (Research Question 2). These findings correspond to earlier literature that reported group differences in these IDS-2 subtests (Schweizer et al., 2021) and in the IDS and IDS-P subtest Auditory Memory (Grob, Meyer, & Hagmann-von Arx, 2013; Grob, Reimann, et al., 2013). We assume that—as we accounted for language abilities—other components of having a linguistically diverse background contributed to the variance in Story Recall and Naming Opposites. Nevertheless, we can provide only assumptions about possible explanations for our results since these were exploratory analyses. One reason for the significant association between bi/multilingualism and Naming Opposites might be that this subtest of Verbal Reasoning measures crystallized components of intelligence according to the CHC model (McGrew, 1997, 2009; Schneider & McGrew, 2018). This includes, besides verbal aspects, “the ability to comprehend and communicate culturally-valued knowledge. . .developed through experience, learning and acculturation” (Schneider & McGrew, 2018, p. 114). Therefore, this subtest might be dependent on factors such as cultural background, socialization, and previous schooling and learning experiences.

The subtest Story Recall assesses verbal Long-Term Memory and encompasses a verbal-dependent presentation and recall format (Schweizer et al., 2021). In addition, the content of the story might also partly contain socialization or culture-specific aspects as it includes names (e.g., Judith, Daniel) that are commonly used in German-speaking countries and elements, such as “inflatable boat” or “oar”, which have to be remembered by the participants. Children from linguistically diverse backgrounds might be less familiar with such names or expressions and therefore may have more difficulties remembering them. However, our results should be considered preliminary, and future research is needed.

In contrast to previous research (e.g., Foy & Mann, 2014; Yurtsever et al., 2023), we did not find any advantage for bi/multilingual children in the executive functions domain. This might be explained by the fact that previous studies found results in favor of bilinguals mainly when examining nonverbal executive functions, whereas the IDS-2 executive function tasks tend to be more dependent on verbal skills, as the associations with language abilities in our study have shown. However, it is important to note that there is a growing body of research that challenges the bilingual advantage hypothesis in general (Lowe et al., 2021; Paap et al., 2016).

### Strengths, Limitations, and Future Directions

One strength of this study is that we relied on a comprehensive test battery and could therefore provide a more complete picture and nuanced exploration of the relative importance of language aspects in test performance in various cognitive and developmental domains compared to previous studies. In line with this, we could also investigate relations at the level of subtests, intelligence group factors, and composites. Moreover, we used standardized assessments of children’s receptive and expressive language ability and included therefore objective measures of proficiency in the test language. We also consider it a main strength that we incorporated multiple language aspects to gain insights regarding their differential contribution to test performance, thus following an integrated approach. In addition, we included a rather large sample that was representative of the percentage of bi/multilingual children in German-speaking countries (German Federal Statistical Office, 2022; Swiss Federal Statistical Office, 2021). We also controlled for sex and SES in the hierarchical regression analyses before entering the language aspects in the model and conducted post-hoc analyses with age as potential moderator, which allowed us to take into account possible confounding effects of relevant sociodemographic characteristics (Weiss & Saklofske, 2020).

Our study also has limitations that should be acknowledged in future research. Given that the associations between language aspects and IDS-2 scores were cross-sectional, it is not possible to draw any conclusions about causal relationships or the direction of effects. Furthermore, our sample included only children aged 5 to 10 years because the subtest Language Skills is not administered to adolescents in the IDS-2. As previous research found age effects in the IDS (Hagmann-von Arx et al., 2013) and in the intelligence domain of the IDS-2 (Schweizer et al., 2021), future research should examine the relations between objectively measured language abilities and test scores on the IDS-2 in adolescents. Although we controlled for SES, as represented by maternal educational background, a larger proportion of the children’s mothers had completed postsecondary education in our study compared to census data of the corresponding population (e.g., Swiss Federal Statistical Office, 2022b), which reduces generalizability. In addition, we had only limited information regarding children’s native languages and therefore could not consider other variables, such as how often the children spoke the test language in the family context or when they were first exposed to the test language and their other language(s), which could be used to examine potential moderating effects of successive and simultaneous bilingualism. In line with this point, future research should collect information on children’s migration status (e.g., country of birth), as we only had broad data on nationality. As the majority of the children in our study attended regular school settings and did not display psychological diagnoses, our findings may not be generalizable to children with developmental risk factors or disorders (e.g., children with autism spectrum disorder). Future studies should therefore investigate the role of language aspects in test performance in the IDS-2 specifically in children with special needs, as these children typically undergo assessments of cognitive and developmental functions in psychological practice. We further encourage future research to focus on samples with culturally diverse individuals (e.g., non-European origin) and include assessments of participants’ level of acculturation in order to control for cultural effects when examining associations between language aspects and IDS-2 test scores. Moreover, studies examining measurement invariance should be conducted to test the equivalence and reliability of the IDS-2 across groups with linguistically diverse samples. Finally, the present findings cannot be extrapolated to other versions of the IDS-2 as evidence on validity needs to be provided for each application of a specific test (American Educational Research Association et al., 2014). Therefore, we encourage researchers to investigate associations between language aspects and test performance in the other language adaptations of the IDS-2.

For test construction, we suggest developing nonverbal tests and test batteries with minimal verbal demands. In such batteries, nonverbal instructions and response options could be implemented through gestures, pictures, and novel digital approaches using sound and video elements. Moreover, dynamic testing could be used to develop tests as it focuses on learning processes in addition to the final performance by including training phases, demonstration by the test administrator, and feedback (Guthke & Wiedl, 1996).

### Implications

When assessing cognitive and developmental functions in bi/multilingual participants, clinicians encounter substantial challenges, such as limited proficiency in the test language, which could mean an individual’s true ability is underestimated (Hagmann-von Arx et al., 2013). According to current guidelines on standards for psychological testing, test administrators should therefore examine the validity of score interpretations for bi/multilingual participants and participants who have reduced proficiency in the test language (American Educational Research Association et al., 2014). In line with this, our study provides evidence to support the claim that particularly information about participants’ language proficiency should be gathered during the diagnostic process (Flanagan et al., 2007), if there are hints that the participant may have linguistic disadvantages in the test language (e.g., assessed as part of the anamnestic evaluation and clinical judgment). By doing this, it would be possible to determine where on the continuum of language proficiency the participants are located, which would lead to a more accurate consideration of possible relations between language abilities and individuals’ test performance (Cormier et al., 2022).

Considering that the IDS-2 even includes a standardized measure of language skills, we therefore suggest that—when there is a suspicion that the participant may have difficulties in the test language—clinicians should assess children’s receptive language abilities prior to the administration of all domains of the IDS-2. We further propose measuring children’s expressive language abilities before administering the IDS-2 intelligence and basic skills domains to participants at risk for linguistic disadvantages. If proficiency in the test language cannot be guaranteed or bi/multilingual participants show, despite sufficient language abilities, considerable difficulties in IDS-2 subtests with high verbal demands, a nonverbal or culture-fair test or a test that relies on the concept of dynamic testing should be conducted. In addition, one of the several language adaptations of the IDS-2 (Grob et al., 2018, 2019, 2021, 2022) could be used if the participant has superior language abilities in that language. If there is no such test battery available for the specific domain, possible linguistic disadvantages have to be considered in the interpretation of the IDS-2 test scores. However, as basic skills represent “cultural skills” that are, for example, related to input from the school environment and therefore reflect previous language experiences (Grob & Hagmann-von Arx, 2018b; Köller & Baumert, 2008), associations between language abilities and performance on the basic skills subtests are to be expected. Hence, the validity of test score interpretations in this domain is not compromised by insufficient test language proficiency. However, participants’ language abilities should still be taken into account in the interpretation of scores derived from the basic skills domain. Finally, although measuring participants’ language abilities in addition to the cognitive or developmental domain that covers the main question of the psychological assessment will result in a somewhat longer duration of the test administration, it is essential to consider children’s proficiency in the test language to draw accurate and valid conclusions for recommendations, treatment, and high-stakes diagnostic decisions.

## Supplemental Material

sj-docx-1-asm-10.1177_10731911251315027 – Supplemental material for The Role of Language Aspects in the Assessment of Cognitive and Developmental Functions in Children: An Analysis of the Intelligence and Development Scales–2 Supplemental material, sj-docx-1-asm-10.1177_10731911251315027 for The Role of Language Aspects in the Assessment of Cognitive and Developmental Functions in Children: An Analysis of the Intelligence and Development Scales–2 by Salome D. Odermatt, Silvia Grieder, Florine Schweizer, Anette Bünger and Alexander Grob in Assessment

## Notes

1. We formed the variable *bi/multilingualism* using information regarding children’s native language: Bi/multilingual children spoke German (the test language) and had at least one other language as their native language, whereas monolingual children indicated German as their native language. As the majority of the multilingual children were bilingual in our sample (see method section), we use the term “bi/multilingualism” to emphasize the pivotal role of bilingualism within this group of children.

2. As we used age-standardized scores, we did not additionally control for age in the hierarchical regression analyses. However, to rule out the possibility of moderating effects of age, we also performed post-hoc analyses to examine whether age moderated the associations between language aspects and scores on the IDS-2. The included interaction terms (i.e., receptive language ability*age, expressive language ability*age, bi/multilingualism*age) were not significant in any of the analyses, indicating that the relations between language aspects and IDS-2 scores were not moderated by age (see Tables S20 to S27 in the Supplement).

3. We postulate that developmental domains are both distinguishable and interrelated. From a clinical perspective, comprehensive assessments of children reveal interindividual and intraindividual strengths and weaknesses in different developmental domains. Furthermore, different developmental domains can be distinguished in correlation analyses when scores are associated less closely with measures of other abilities (e.g., in analyses of discriminant validity evidence; American Educational Research Association et al., 2014). Nevertheless, already one of the first constructivist views on development by Binet (1909) argued that developmental domains are also interrelated. In the context of assessment, high correlations between domains may be attributed to the interrelatedness of the underlying constructs. For example, associations between language abilities and literacy skills (e.g., Storch & Whitehurst, 2002) or between language abilities and Verbal Reasoning (Schneider & McGrew, 2018) are expected, as language abilities are considered to be a component of the construct. Moreover, certain tasks of tests necessitate the performance of additional skills, which means that test results can be related to other processes that are not or only partially part of the construct (i.e., the problem of task impurity). For example, to complete a task, it may be necessary to give a verbal response and, hence, language abilities may be related to performance on that task. The problem of task impurity may indicate that language abilities are more crucial for the assessment of other developmental domains than vice versa. For instance, it is rather unlikely that intelligence is also assessed when measuring language abilities, given that the content of the tasks to assess language abilities is typically not complex. Nevertheless, it is important to acknowledge that we are not claiming that there is a unidirectional pattern in the development of domains. Moreover, our data are cross-sectional, which precludes any inferences about the direction of the relationship.

4. In this sample, the following medical and psychological diagnoses were reported: visual problems (*n* = 59), hearing problems (*n* = 6), motor problems (*n* = 16), speech problems (*n* = 16), giftedness (*n* = 21), intellectual disabilities (*n* = 19), scholastic problems (*n* = 15), behavioral problems (*n* = 20), autism spectrum disorder (*n* = 5), affective symptoms (*n* = 2), physical problems (*n* = 28), and other (*n* = 2).

5. As the minimum requirement for participation in the IDS-2 testing is to understand instructions and questions and, thus, receptive language ability, we entered this language aspect first. In addition, as receptive language ability precedes expressive language ability, we added expressive language ability as second language predictor. Children are able to understand words before they can use them (Grimm, 2012), and it is evident that it is not possible to speak a language when one does not comprehend it. Finally, as bi/multilingualism may encompass other aspects beyond those measured in language abilities, we entered this variable as third language aspect to detect possible associations in addition to receptive and expressive language abilities. Moreover, we used forward-selection to include the language aspects.

6. We did not perform analyses for the basic skills composite because receptive and expressive language abilities are also contained in the calculation of this score. Moreover, we did not examine the IDS-2 domain motivation and attitude as its subtests are administered only to adolescents aged 11 to 20 years.

7. We also conducted post-hoc Pearson correlation analyses between the language aspects and the IDS-2 scores. Results demonstrated that receptive and expressive language abilities were related to all scores on the IDS-2. Bi/multilingualism showed associations with the three intelligence composites, four intelligence group factors (i.e., Auditory Short-Term Memory, Abstract Reasoning, Verbal Reasoning, Long-Term Memory), seven intelligence subtests (i.e., Digit and Letter Span, Mixed Digit and Letter Span, Matrices: Completion, Naming Categories, Naming Opposites, Story Recall, Picture Recall), the executive functions composite and two subtests (i.e., Listing Words, Divided Attention), and the three basic skills subtests (i.e., Logical-Mathematical Reasoning, Reading, Spelling; see Tables S3 to S7 in the Supplement).

8. To control for effects of intelligence, we repeated the hierarchical regression analyses with intelligence (Profile IQ) included as a predictor of the IDS-2 domains executive functions, psychomotor skills, social-emotional skills, and basic skills. Although children’s language abilities were no longer associated with many of the IDS-2 scores after accounting for intelligence, the overall pattern of results remained largely the same, as receptive and expressive language abilities were still particularly related to the subtests of the basic skills domain (see Tables S16 to S19 in the Supplement for full results). However, as the Profile IQ is highly correlated with language abilities (*r* = .50–.60; see Table 3), these findings need to be treated with caution and further research should use a nonverbal intelligence assessment when accounting for intelligence effects in this context.

## References

[bibr1-10731911251315027] Abutalebi J. Green D. W. (2008). Control mechanisms in bilingual language production: Neural evidence from language switching studies. Language and Cognitive Processes, 23(4), 557–582. 10.1080/01690960801920602

[bibr2-10731911251315027] American Educational Research Association, American Psychological Association, & National Council on Measurement in Education. (2014). Standards for educational and psychological testing.

[bibr3-10731911251315027] American Psychological Association. (2015). APA dictionary of psychology (2nd ed.).

[bibr4-10731911251315027] Austrian Federal Statistical Office. (2022). Mehr als ein Viertel der österreichischen Gesamtbevölkerung hat Migrationshintergrund [More than a quarter of the total Austrian population has a migration background]. https://www.statistik.at/web_de/statistiken/menschen_und_gesellschaft/bevoelkerung/bevoelkerungsstruktur/bevoelkerung_nach_migrationshintergrund/033240.html

[bibr5-10731911251315027] Baltes P. B. (1987). Theoretical propositions of life-span developmental psychology: On the dynamics between growth and decline. Developmental Psychology, 23(5), 611–626.

[bibr6-10731911251315027] Baltes P. B. (1990). Entwicklungspsychologie der Lebensspanne: Theoretische Leitsätze [Lifespan developmental psychology: Guiding theoretical principles]. Psychologische Rundschau, 41, 1–24.

[bibr7-10731911251315027] Barac R. Bialystok E. Castro D. C. Sanchez M. (2014). The cognitive development of young dual language learners: A critical review. Early Childhood Research Quarterly, 29(4), 699–714. 10.1016/j.ecresq.2014.02.003 25284958 PMC4180217

[bibr8-10731911251315027] Bialystok E. Craik F. I. M. (2010). Cognitive and linguistic processing in the bilingual mind. Current Directions in Psychological Science, 19(1), 19–23. 10.1177/0963721409358571

[bibr9-10731911251315027] Bialystok E. Luk G. Peets K. F. Yang S. (2010). Receptive vocabulary differences in monolingual and bilingual children. Bilingualism: Language and Cognition, 13(4), 525–531. 10.1017/S1366728909990423 25750580 PMC4349351

[bibr10-10731911251315027] Binet A. (1909). Les idées modernes sur les enfants. Flammarion.

[bibr11-10731911251315027] Bos W. Lankes E.-M. Prenzel M. Schwippert K. Valtin R. Walther G. (2007). Erste Ergebnisse aus IGLU: Schülerleistungen am Ende der vierten Jahrgangsstufe im internationalen Vergleich [First results from IGLU: Student performance at the end of the fourth grade in international comparison]. Sozialwissenschaftlicher Fachinformationsdienst SoFid, Bildungsforschung, 2007(1), 9–46.

[bibr12-10731911251315027] Calero M. D. Fernández-Parra A. López-Rubio S. Carles R. Mata S. del Carmen Vives M. Navarro E. Márquez J. (2013). Variables involved in personal, social and school adjustment in a sample of preschool-aged children from different cultural backgrounds. European Journal of Psychology of Education, 28(1), 133–155. 10.1007/s10212-012-0107-8

[bibr13-10731911251315027] Cormier D. C. Bulut O. McGrew K. S. Kennedy K. (2022). Linguistic influences on cognitive test performance: Examinee characteristics are more important than test characteristics. Journal of Intelligence, 10(1), Article 1. 10.3390/jintelligence10010008 PMC8883963 35225924

[bibr14-10731911251315027] Cormier D. C. McGrew K. S. Evans J. J. (2011). Quantifying the “degree of linguistic demand” in spoken intelligence test directions. Journal of Psychoeducational Assessment, 29(6), 515–533. 10.1177/0734282911405962

[bibr15-10731911251315027] Daseking M. Lipsius M. Petermann F. Waldmann H.-C. (2008). Differenzen im Intelligenzprofil bei Kindern mit Migrationshintergrund: Befunde zum HAWIK-IV [Differences in the intelligence profile of children with a migration background: Findings from the HAWIK-IV]. Kindheit und Entwicklung, 17(2), 76–89. 10.1026/0942-5403.17.2.76

[bibr16-10731911251315027] Daseking M. Pauls F. Petermann F. Becker A. (2018). Welchen Einfluss hat ein Migrationshintergrund auf die kognitiven Leistungen in der WISC-V? [What is the effect of migration background on cognitive performance on the WISC-V?]. Kindheit und Entwicklung, 27(3), 175–183. 10.1026/0942-5403/a000257

[bibr17-10731911251315027] Ehm J.-H. Duzy D. Hasselhorn M. (2011). Das akademische Selbstkonzept bei Schulanfängern [The academic self-concept of pupils at the beginning of school]. Frühe Bildung, 0(0), 37–45. 10.1026/2191-9186/a000008

[bibr18-10731911251315027] Flanagan D. P. Harrison P. L. (2012). Contemporary intellectual assessment: Theories, tests, and issues (3rd ed.). Guilford Press.

[bibr19-10731911251315027] Flanagan D. P. Ortiz S. O. (2001). Essentials of cross-battery assessment. Wiley.

[bibr20-10731911251315027] Flanagan D. P. Ortiz S. O. Alfonso V. C. (2007). Essentials of cross-battery assessment with C/D ROM (2nd ed.). Wiley.

[bibr21-10731911251315027] Foy J. G. Mann V. A. (2014). Bilingual children show advantages in nonverbal auditory executive function task. International Journal of Bilingualism, 18(6), 717–729. 10.1177/1367006912472263

[bibr22-10731911251315027] German Federal Statistical Office. (2022). Statistiken der Kinder- und Jugendhilfe [Statistics of child and youth services]. https://www.destatis.de/DE/Themen/Gesellschaft-Umwelt/Soziales/Kindertagesbetreuung/Publikationen/Downloads-Kindertagesbetreuung/tageseinrichtungen-kindertagespflege-5225402227004.pdf

[bibr23-10731911251315027] German Federal Statistical Office. (2023). Bevölkerung nach Migrationshintergrund und Geschlecht [Population by migration background and gender]. https://www.destatis.de/DE/Themen/Gesellschaft-Umwelt/Bevoelkerung/Migration-Integration/Tabellen/liste-migrationshintergrund-geschlecht.html

[bibr24-10731911251315027] Ginsburg H. P. (1997). Entering the child’s mind. Cambridge University Press.

[bibr25-10731911251315027] Goldstein S. Princiotta D. Naglieri J. A. (2015). Handbook of intelligence: Evolutionary theory, historical perspective, and current concepts. Springer.

[bibr26-10731911251315027] Grieder S. Bünger A. Odermatt S. D. Schweizer F. Grob A. (2022). Limited internal comparability of general intelligence composites: Impact on external validity, possible predictors, and practical remedies. Assessment, 29(6), 1172–1189. 10.1177/10731911211005171 33794710 PMC9301611

[bibr27-10731911251315027] Grieder S. Grob A. (2020). Exploratory factor analyses of the Intelligence and Development Scales–2: Implications for theory and practice. Assessment, 27(8), 1853–1869. 10.1177/1073191119845051 31023061

[bibr28-10731911251315027] Grimm H. (2012). Störungen der Sprachentwicklung: Grundlagen—Ursachen—Diagnose—Intervention—Prävention [Language development disorders: Basics—causes—diagnosis—intervention—prevention] (3rd ed.). Hogrefe.

[bibr29-10731911251315027] Grob A. Hagmann-von Arx P. (2018a). Intelligence and Development Scales–2 (IDS-2). Intelligenz- und Entwicklungsskalen für Kinder und Jugendliche [Intelligence and Development Scales for Children and Adolescents]. Hogrefe.

[bibr30-10731911251315027] Grob A. Hagmann-von Arx P. (2018b). Intelligence and Development Scales–2 (IDS-2). Intelligenz- und Entwicklungsskalen für Kinder und Jugendliche. Manual zu Theorie, Interpretation und Gütekriterien [Intelligence and Development Scales for Children and Adolescents. Manual on theory, interpretation and psychometric criteria]. Hogrefe.

[bibr31-10731911251315027] Grob A. Hagmann-von Arx P. Barnett A. Stuart N. Vanzan S. (2021). Intelligence and Development Scales–2 (IDS-2). Intelligence and Development Scales for Children and Adolescents. Hogrefe.

[bibr32-10731911251315027] Grob A. Hagmann-von Arx P. Ferri R. Rea M. Casagrande M. (2022). Intelligence and Development Scales–2 (IDS-2). Scale di intelligenza e sviluppo per bambini e adolescenti [Intelligence and Development Scales for Children and Adolescents]. Hogrefe.

[bibr33-10731911251315027] Grob A. Hagmann-von Arx P. Jaworowska A. Matczak A. Fecenec D. (2019). Intelligence and Development Scales–2 (IDS-2). Inteligencji i Rozwoju dla Dzieci i Młodzieży [Intelligence and Development Scales for Children and Adolescents]. Hogrefe.

[bibr34-10731911251315027] Grob A. Hagmann-von Arx P. Ruiter S. A. J. Timmerman M. E. Visser L. (2018). Intelligence and Development Scales–2 (IDS-2). Intelligentie- en ontwikkelingsschalen voor kinderen en jongeren [Intelligence and Development Scales for Children and Adolescents]. Hogrefe.

[bibr35-10731911251315027] Grob A. Keller K. Troesch L. M. (2014). ZWEITSPRACHE–Mit ausreichenden Deutschkenntnissen in den Kindergarten [Second language–with sufficient German skills in kindergarten]. Hogrefe.

[bibr36-10731911251315027] Grob A. Meyer C. S. Hagmann-von Arx P. (2009). Intelligence and Development Scales (IDS). Huber. 10.1024/1422-4917/a000148 22161941

[bibr37-10731911251315027] Grob A. Meyer C. S. Hagmann-von Arx P. (2013). Intelligence and Development Scales (IDS): Intelligenz- und Entwicklungsskalen für Kinder von 5–10 Jahren [Intelligence and Development Scales for Children 5–10 years old]. Hans Huber.

[bibr38-10731911251315027] Grob A. Reimann G. Gut J. Frischknecht M.-C. (2013). Intelligence and Development Scales–Preschool (IDS-P): Intelligenz- und Entwicklungsskalen für das Vorschulalter [Intelligence and Development Scales for Preschool Children]. Hans Huber.

[bibr39-10731911251315027] Grundy J. G. (2020). The effects of bilingualism on executive functions: An updated quantitative analysis. Journal of Cultural Cognitive Science, 4(2), 177–199. 10.1007/s41809-020-00062-5

[bibr40-10731911251315027] Guthke J. Wiedl K. H. (1996). Dynamisches Testen: Zur Psychodiagnostik der intraindividuellen Variabilität [Dynamic testing: On the psychodiagnostics of intraindividual variability]. Hogrefe.

[bibr41-10731911251315027] Hagmann-von Arx P. Petermann F. Grob A . (2013). Konvergente und diskriminante Validität der WISC-IV und der Intelligence and Development Scales (IDS) bei Kindern mit Migrationshintergrund [Convergent and discriminant validity of the WISC-IV and the Intelligence and Development Scales (IDS) in children with a migration background]. Diagnostica, 59(4), 170–182. 10.1026/0012-1924/a000091

[bibr42-10731911251315027] Hessels M. (1997). Low IQ but high learning potential: Why Zeyneb and Moussa do not belong in special education. Educational and Child Psychology, 14, 121–136.

[bibr43-10731911251315027] Hoff E. Core C. Place S. Rumiche R. Señor M. Parra M. (2012). Dual language exposure and early bilingual development. Journal of Child Language, 39(1), 1–27. 10.1017/S0305000910000759 21418730 PMC4323282

[bibr44-10731911251315027] Hommel G. (1988). A stagewise rejective multiple test procedure based on a modified Bonferroni test. Biometrika, 75, 383–386.

[bibr45-10731911251315027] Kastner J. W. May W. Hildman L. (2001). Relationship between language skills and academic achievement in first grade. Perceptual and Motor Skills, 92(2), 381–390. 10.2466/pms.2001.92.2.381 11361297

[bibr46-10731911251315027] Kauschke C. (2012). Kindlicher Spracherwerb im Deutschen: Verläufe, Forschungsmethoden, Erklärungsansätze [Children’s language acquisition in German: Trajectories, research methods, explanatory approaches]. Walter de Gruyter.

[bibr47-10731911251315027] Klingner J. K. Blanchett W. J. Harry B. (2007). Race, culture, and developmental disabilities. In Odom S. L. Horner R. H. Snell M. E. Blacher J. (Eds.), Handbook of developmental disabilities (pp. 55–75). Guilford Press.

[bibr48-10731911251315027] Köller O. Baumert J. (2008). Entwicklung schulischer Leistungen [Development of scholastic performance]. In Oerter R. Montada L. (Eds.), Entwicklungspsychologie [Developmental psychology] (Vol. 6) (pp. 735–768). Beltz. https://pure.mpg.de/pubman/faces/ViewItemOverviewPage.jsp?itemId=item_2102046

[bibr49-10731911251315027] Konsortium PISA.ch. (2018). PISA 2015: Schülerinnen und Schüler der Schweiz im internationalen Vergleich [PISA 2015: Swiss students in international comparison]. SBFI/EDK und Konsortium PISA.ch.

[bibr50-10731911251315027] Konsortium PISA.ch. (2019). PISA 2018: Schülerinnen und Schüler der Schweiz im internationalen Vergleich [PISA 2018: Swiss students in international comparison]. SBFI/EDK and Konsortium PISA.ch.

[bibr51-10731911251315027] Kroll J. F. Dussias P. E. Bogulski C. A. Kroff J. R. V. (2012). Chapter seven—Juggling two languages in one mind: What bilinguals tell us about language processing and its consequences for cognition. In Ross B. H. (Ed.), Psychology of learning and motivation (Vol. 56) (pp. 229–262). Academic Press. 10.1016/B978-0-12-394393-4.00007-8

[bibr52-10731911251315027] Lee Swanson H. Rosston K. Gerber M. Solari E . (2008). Influence of oral language and phonological awareness on children’s bilingual reading. Journal of School Psychology, 46(4), 413–429. 10.1016/j.jsp.2007.07.002 19083366

[bibr53-10731911251315027] Lienert G. A. Raatz U. (1998). Testaufbau und Testanalyse [Test design and test analysis]. Beltz.

[bibr54-10731911251315027] Lowe C. J. Cho I. Goldsmith S. F. Morton J. B. (2021). The bilingual advantage in children’s executive functioning is not related to language status: A meta-analytic review. Psychological Science, 32(7), 1115–1146. 10.1177/0956797621993108 34213379 PMC8641133

[bibr55-10731911251315027] McGrew K. S. (1997). Analysis of the major intelligence batteries according to a proposed comprehensive Gf-Gc framework. In Flanagan D. P. Genshaft J. L. Harrison P. L. (Eds.), Contemporary intellectual assessment: Theories, tests, and issues (pp. 151–119). Guilford Press.

[bibr56-10731911251315027] McGrew K. S. (2009). CHC theory and the human cognitive abilities project: Standing on the shoulders of the giants of psychometric intelligence research. Intelligence, 37(1), 1–10. 10.1016/j.intell.2008.08.004

[bibr57-10731911251315027] Melby-Lervåg M. Lervåg A. (2014). Reading comprehension and its underlying components in second-language learners: A meta-analysis of studies comparing first- and second-language learners. Psychological Bulletin, 140(2), 409–433. 10.1037/a0033890 23937316

[bibr58-10731911251315027] Odermatt S. D. Möhring W. Grieder S. Grob A. (2022). Cognitive and developmental functions in autistic and non-autistic children and adolescents: Evidence from the Intelligence and Development Scales–2. Journal of Intelligence, 10(4), Article 4. 10.3390/jintelligence10040112 PMC9680381 36412792

[bibr59-10731911251315027] Ortiz S. O. (2019). On the measurement of cognitive abilities in English learners. Contemporary School Psychology, 23(1), 68–86. 10.1007/s40688-018-0208-8

[bibr60-10731911251315027] Ouellette G. P. (2006). What’s meaning got to do with it: The role of vocabulary in word reading and reading comprehension. Journal of Educational Psychology, 98(3), 554–566. 10.1037/0022-0663.98.3.554

[bibr61-10731911251315027] Paap K. R. Johnson H. A. Sawi O. (2016). Should the search for bilingual advantages in executive functioning continue? Cortex, 74, 305–314. 10.1016/j.cortex.2015.09.010 26586100

[bibr62-10731911251315027] Paetsch J. Radmann S. Felbrich A. Lehmann R. Stanat P. (2016). Sprachkompetenz als Prädiktor mathematischer Kompetenzentwicklung von Kindern deutscher und nicht-deutscher Familiensprache [Language competence as a predictor of mathematical competence development of children of German and non-German family language]. Zeitschrift für Entwicklungspsychologie und Pädagogische Psychologie, 48(1), 27–41. 10.1026/0049-8637/a000142

[bibr63-10731911251315027] Petermann F. (Ed.) (2017). Wechsler Intelligence Scale for Children—Fifth Edition (WISC–V). German Adaptation. Pearson.

[bibr64-10731911251315027] Petermann F. Petermann U. (2011). Wechsler Intelligence Scale for Children—Fourth Edition (WISC–IV). German Adaptation. Pearson.

[bibr65-10731911251315027] R Core Team. (2022). R: A language and environment for statistical computing (Version 2022.12.0+353)[Computer software]. R Foundation for Statistical Computing. https://www.R-project.org/

[bibr66-10731911251315027] Reich H. H. Roth H.-J. Dirim I. Jørgensen J. N. List G. List G. Neumann U. Siebert-Ott G. Steinmüller U. Teunissen F. Vallen T. Wurnig V. (2002). Spracherwerb zweisprachig aufwachsender Kinder und Jugendlicher: Ein Überblick über den Stand der nationalen und internationalen Forschung [Language acquisition of children and adolescents growing up bilingually: An overview of the state of national and international research]. Freie und Hansestadt Hamburg, Behörde für Bildung und Sport.

[bibr67-10731911251315027] Rodriguez-Fornells A. Rotte M. Heinze H.-J. Nösselt T. Münte T. F. (2002). Brain potential and functional MRI evidence for how to handle two languages with one brain. Nature, 415(6875), Article 6875. 10.1038/4151026a 11875570

[bibr68-10731911251315027] Schneider W. J. McGrew K. S. (2018). The Cattell–Horn–Carroll theory of cognitive abilities. In Flanagan D. P. McDonough E. M. (Eds.), Contemporary intellectual assessment: Theories, tests, and issues (4th ed.) (pp. 73–163). Guilford Press.

[bibr69-10731911251315027] Schrank F. A. McGrew K. S. Mather N. (2014). Woodcock—Johnson IV tests of cognitive abilities. Riverside Publishing.

[bibr70-10731911251315027] Schweizer F. Grieder S. Bünger A. Grob A. (2021). Vergleich von Intelligenztestleistungen bei monolingualen und bilingualen Kindern und Jugendlichen in den Intelligence and Development Scales–2 (IDS-2) [Comparison of intelligence test performance of monolingual and bilingual children and adolescents on the Intelligence and Development Scales-2 (IDS-2)]. Diagnostica, 67(1), 36–46. 10.1026/0012-1924/a000260

[bibr71-10731911251315027] Storch S. A. Whitehurst G. J. (2002). Oral language and code-related precursors to reading: Evidence from a longitudinal structural model. Developmental Psychology, 38(6), 934–947. 10.1037/0012-1649.38.6.934 12428705

[bibr72-10731911251315027] Sullivan A. L. (2011). Disproportionality in special education identification and placement of English language learners. Exceptional Children, 77(3), 317–334. 10.1177/001440291107700304

[bibr73-10731911251315027] Swiss Federal Statistical Office. (2021). Erhebung zur Sprache, Religion und Kultur 2019 [Language, religion, and culture survey 2019]. https://www.bfs.admin.ch/bfs/de/home/statistiken/kataloge-datenbanken/medienmitteilungen.assetdetail.15384140.html

[bibr74-10731911251315027] Swiss Federal Statistical Office. (2022a). Bevölkerung nach Migrationsstatus [Population by migration status]. https://www.bfs.admin.ch/bfs/de/home/statistiken/bevoelkerung/migration-integration/nach-migrationsstatuts.html

[bibr75-10731911251315027] Swiss Federal Statistical Office. (2022b). Bildungsstand der Wohnbevölkerung nach Alter und Geschlecht [Educational attainment of the resident population by age and gender]. https://www.bfs.admin.ch/bfs/de/home/statistiken/kataloge-datenbanken/tabellen.assetdetail.22024469.html

[bibr76-10731911251315027] Thierry G. Wu Y. J. (2007). Brain potentials reveal unconscious translation during foreign-language comprehension. Proceedings of the National Academy of Sciences, 104(30), 12530–12535. 10.1073/pnas.0609927104 PMC1941503 17630288

[bibr77-10731911251315027] United Nations. (2022). World migration report 2022. https://worldmigrationreport.iom.int/wmr-2022-interactive/

[bibr78-10731911251315027] Verhoeven L. (2000). Components in early second language reading and spelling. Scientific Studies of Reading, 4(4), 313–330. 10.1207/S1532799XSSR0404_4

[bibr79-10731911251315027] Weiss L. G. Harris J. G. Prifitera A. Courville T. Rolfhus E. Saklofske D. H. Holdnack J. A. (2006). WISC-IV interpretation in societal context. The essentials and beyond. In Weiss L. G. Saklofske D. H. Prifitera A. Holdnack J. A. (Eds.), WISC-IV: Advanced clinical interpretation (pp. 1–49). Elsevier.

[bibr80-10731911251315027] Weiss L. G. Saklofske D. H. (2020). Mediators of IQ test score differences across racial and ethnic groups: The case for environmental and social justice. Personality and Individual Differences, 161, Article 109962. 10.1016/j.paid.2020.109962

[bibr81-10731911251315027] Yurtsever A. Anderson J. A. E. Grundy J. G. (2023). Bilingual children outperform monolingual children on executive function tasks far more often than chance: An updated quantitative analysis. Developmental Review, 69, Article 101084. 10.1016/j.dr.2023.101084

