# Supplementary material for: The Role of Language Aspects in the Assessment of Cognitive and Developmental Functions in Children: An Analysis of the Intelligence and Development Scales–2
Source: Assessment. 2025 Feb 3;33(1):27–45. doi: 10.1177/10731911251315027 (PMC12686191; doi:10.1177/10731911251315027)
Supplement: sj-docx-1-asm-10.1177_10731911251315027 – Supplemental material for The Role of Language Aspects in the Assessment of Cognitive and Developmental Functions in Children: An Analysis of the Intelligence and Development Scales–2 [file sj-docx-1-asm-10.1177_10731911251315027.docx]

**Supplementary Material to “The Role of Language Aspects in the Assessment of Cognitive and Developmental Functions in Children: An Analysis of the Intelligence and Development Scales–2”**

**Table S1**

*Description of the Domains of the Intelligence and Development Scales–2 (IDS-2) Included in Our Study*

| Domain | Group factor | Subtest | No. of items | Description |
| --- | --- | --- | --- | --- |
| Intelligence | Visual Processing (VP) | Shape Design (SD) | 20 | Reproduce geometric figures with the help of rectangles and triangles |
|  |  | Washer Design (WD) | 2–4^a^ | Reproduce counter patterns according to a template |
|  | Processing Speed (PS) | Parrots (PSP) | 56–180^a^ | Cross out parrots with two orange features that look to the left from rows of different parrots |
|  |  | Boxes (PSB) | 104–180^a^ | Cross out groups of three or four boxes from rows of different groups of boxes |
|  | Auditory Short-Term Memory (ASTM) | Digit and Letter Span (DLS) | 40 | Repeat number and letter sequences forward and backward |
|  |  | Mixed Digit and Letter Span (MDLS) | 36 | Repeat mixed number and letter sequences forward and backward |
|  | Visuospatial Short-Term Memory (VSTM) | Shape Memory (SM) | 23 | Remember figures and recognize them from a selection of figures and positions |
|  |  | Rotated Shape Memory (RSM) | 23 | Remember figures and recognize them from a selection of rotated figures and positions |
|  | Abstract Reasoning (AR) | Matrices: Completion (MC) | 35 | Understand how a figure changes and transfer these changes to a continuing figure |
|  |  | Matrices: Odd One Out (MOO) | 31 | Select from presented pictures the one that does not fit with the others |
|  | Verbal Reasoning (VR) | Naming Categories (NC) | 34 | Name categories for a group of pictures or words |
|  |  | Naming Opposites (NO) | 34 | Name opposites of presented words |
|  | Long-Term Memory (LTM) | Story Recall (SR) | 19–32^a^ | Listen to a semantically meaningful story and recall it after at least 20 min |
|  |  | Picture Recall (PR) | 11–21^a^ | Look at a picture and recall key features and details after at least 20 min |
| Executive functions |  | Listing Words (LW) | 2-4^a^ | List words based on categories or starting letters |
|  |  | Divided Attention (DA) | 50-100^a^ | Cross out parrots with two orange features that look to the left from different parrots and list animals |
|  |  | Animal Colors (AC) | 3 | Say colors of animals as fast as possible |
|  |  | Drawing Routes (DR) | 14 | Travel given routes as fast as possible once |
| Psychomotor skills |  | Gross Motor Skills (GM) | 3 | Balance on a rope, catch and throw a ball, and jump sideways over a rope |
|  |  | Fine Motor Skills (FM) | 6 | Quickly screw nuts on and off bolts of different sizes and quickly thread beads of different sizes |
|  |  | Visuomotor Skills (VM) | 12 | Move exactly between lines, draw figures, and reflect figures |
| Social-emotional skills |  | Identifying Emotions (IE) | 10 | Recognize and name emotions of children in photos |
|  |  | Regulating Emotions (RE) | 6-9^a^ | Specify regulation strategies for the emotions of anger, fear, and grief |
|  |  | Socially Competent Behavior (SC) | 6-9^a^ | Name socially competent behavior according to a presented social situation |
| Basic skills |  | Logical-Mathematical Reasoning (MR) | 64 | Solve logical-mathematical reasoning tasks |
|  |  | Expressive Language Ability (ELA) | 12 | Form sentences from several words |
|  |  | Receptive Language Ability (RLA) | 15 | Carry out instructions |
|  |  | Reading (RD) | 3 | Including reading words, reading pseudo words, and text comprehension |
|  |  | Spelling (SP) | 40-60^a^ | Word dictation |

*Note.* The intelligence domain includes three intelligence composites: (1) Profile IQ (PrIQ) is based on all 14 intelligence subtests, (2) Full-Scale IQ (FSIQ) is based on one subtest from every intelligence group factor (i.e., Shape Design, Parrots, Digit and Letter Span, Shape Memory, Matrices: Completion, Naming Categories, Story Recall), and (3) Screening IQ (ScrIQ) is based on one subtest from the intelligence group factors Abstract Reasoning (i.e., Matrices: Completion) and Verbal Reasoning (i.e., Naming Categories). Reading and Spelling only for ages 7 to 20 years. The domain motivation and attitude is not shown as it is only for ages 11 to 20 years and was therefore omitted in our analyses.

^a^ Depending on age and skill.

**Table S2**

*Characteristics of Monolingual and Bi/Multilingual Children*

| Characteristic | Monolingual  *N* = 611 | | | | Bi/Multilingual  *N* = 215 | | | | *t* | χ^2^ | *p* |
| --- | --- | --- | --- | --- | --- | --- | --- | --- | --- | --- | --- |
|  | *M* | *SD* | *n* | *%* | *M* | *SD* | *n* | *%* |  |  |  |
| Age (years) | 8.09 | 1.67 |  |  | 7.98 | 1.64 |  |  | -0.81 |  | .418 |
| Sex |  |  |  |  |  |  |  |  |  | 117.89 | 1.000 |
| Female |  |  | 322 | 53% |  |  | 102 | 47% |  |  |  |
| Male |  |  | 289 | 47% |  |  | 113 | 53% |  |  |  |
| SES (maternal education) |  |  |  |  |  |  |  |  |  | 126.90 | 1.000 |
| No postsecondary education |  |  | 371 | 61% |  |  | 121 | 56% |  |  |  |
| Postsecondary education |  |  | 240 | 39% |  |  | 94 | 44% |  |  |  |
| Receptive language ability | 10.69 | 2.81 |  |  | 9.32 | 3.26 |  |  | -5.47 |  | **< .001** |
| Expressive language ability | 10.70 | 2.85 |  |  | 9.10 | 3.40 |  |  | -6.17 |  | **< .001** |

*Note.* SES = socioeconomic status. Independent-samples *t* tests for age, receptive language ability, and expressive language ability. χ^2^ test for sex and SES. Significant results are presented in bold.

**Table S3**

*Post-Hoc Pearson Correlations of the Language Aspects and Scores of the Intelligence Domain From the Intelligence and Development Scales–2*

| Variable | | 1 | 2 | 3 | 4 | 5 | 6 | 7 | 8 | 9 | 10 | 11 | 12 | 13 | 14 | 15 | 16 | 17 | 18 | 19 | 20 | 21 | 22 | 23 | 24 | 25 | 26 |
| --- | --- | --- | --- | --- | --- | --- | --- | --- | --- | --- | --- | --- | --- | --- | --- | --- | --- | --- | --- | --- | --- | --- | --- | --- | --- | --- | --- |
| 1 | RLA | — |  |  |  |  |  |  |  |  |  |  |  |  |  |  |  |  |  |  |  |  |  |  |  |  |  |
| 2 | ELA | .45*** | — |  |  |  |  |  |  |  |  |  |  |  |  |  |  |  |  |  |  |  |  |  |  |  |  |
| 3 | BML | .20*** | .23*** | — |  |  |  |  |  |  |  |  |  |  |  |  |  |  |  |  |  |  |  |  |  |  |  |
| 4 | PrIQ | .60*** | .50*** | .16*** | — |  |  |  |  |  |  |  |  |  |  |  |  |  |  |  |  |  |  |  |  |  |  |
| 5 | FSIQ | .58*** | .49*** | .15*** | .95*** | — |  |  |  |  |  |  |  |  |  |  |  |  |  |  |  |  |  |  |  |  |  |
| 6 | ScrIQ | .51*** | .46*** | .13*** | .77*** | .83*** | — |  |  |  |  |  |  |  |  |  |  |  |  |  |  |  |  |  |  |  |  |
| 7 | VP | .35*** | .30*** | .05 | .68*** | .64*** | .47*** | — |  |  |  |  |  |  |  |  |  |  |  |  |  |  |  |  |  |  |  |
| 8 | PS | .33*** | .25*** | .04 | .64*** | .61*** | .37*** | .37*** | — |  |  |  |  |  |  |  |  |  |  |  |  |  |  |  |  |  |  |
| 9 | ASTM | .43*** | .38*** | .11* | .70*** | .66*** | .48*** | .38*** | .34*** | — |  |  |  |  |  |  |  |  |  |  |  |  |  |  |  |  |  |
| 10 | VSTM | .34*** | .27*** | .07 | .64*** | .61*** | .42*** | .35*** | .32*** | .33*** | — |  |  |  |  |  |  |  |  |  |  |  |  |  |  |  |  |
| 11 | AR | .42*** | .36*** | .07* | .74*** | .70*** | .76*** | .45*** | .38*** | .44*** | .42*** | — |  |  |  |  |  |  |  |  |  |  |  |  |  |  |  |
| 12 | VR | .57*** | .52*** | .24*** | .74*** | .74*** | .77*** | .40*** | .34*** | .48*** | .39*** | .45*** | — |  |  |  |  |  |  |  |  |  |  |  |  |  |  |
| 13 | LTM | .41*** | .32*** | .20*** | .63*** | .58*** | .44*** | .31*** | .27*** | .36*** | .28*** | .37*** | .48*** | — |  |  |  |  |  |  |  |  |  |  |  |  |  |
| 14 | SD | .38*** | .28*** | .03 | .67*** | .70*** | .49*** | .83*** | .40*** | .37*** | .39*** | .45*** | .45*** | .29*** | — |  |  |  |  |  |  |  |  |  |  |  |  |
| 15 | WD | .20*** | .21*** | .05 | .47*** | .36*** | .29*** | .82*** | .21*** | .26*** | .19*** | .29*** | .21*** | .23*** | .36*** | — |  |  |  |  |  |  |  |  |  |  |  |
| 16 | PSP | .31*** | .25*** | .06 | .56*** | .59*** | .34*** | .30*** | .87*** | .30*** | .27*** | .33*** | .32*** | .27*** | .31*** | .18*** | — |  |  |  |  |  |  |  |  |  |  |
| 17 | PSB | .27*** | .18*** | .01 | .56*** | .48*** | .30*** | .35*** | .88*** | .29*** | .30*** | .34*** | .28*** | .21*** | .39*** | .20*** | .53*** | — |  |  |  |  |  |  |  |  |  |
| 18 | DLS | .41*** | .36*** | .11** | .68*** | .67*** | .47*** | .38*** | .34*** | .93*** | .31*** | .44*** | .47*** | .33*** | .38*** | .25*** | .30*** | .30*** | — |  |  |  |  |  |  |  |  |
| 19 | MDLS | .39*** | .35*** | .08* | .63*** | .55*** | .42*** | .33*** | .29*** | .93*** | .30*** | .38*** | .42*** | .34*** | .31*** | .23*** | .26*** | .24*** | .73*** | — |  |  |  |  |  |  |  |
| 20 | SM | .28*** | .20*** | .06 | .54*** | .58*** | .36*** | .30*** | .27*** | .27*** | .89*** | .34*** | .32*** | .23*** | .34*** | .16*** | .22*** | .25*** | .28*** | .23*** | — |  |  |  |  |  |  |
| 21 | RSM | .31*** | .28*** | .06 | .56*** | .47*** | .38*** | .30*** | .29*** | .30*** | .83*** | .39*** | .35*** | .27*** | .33*** | .17*** | .24*** | .28*** | .26*** | .29*** | .48*** | — |  |  |  |  |  |
| 22 | MC | .36*** | .34*** | .07* | .64*** | .68*** | .84*** | .42*** | .34*** | .39*** | .36*** | .84*** | .40*** | .31*** | .42*** | .29*** | .30*** | .29*** | .38*** | .35*** | .30*** | .33*** | — |  |  |  |  |
| 23 | MOO | .33*** | .26*** | .04 | .58*** | .47*** | .42*** | .32*** | .30*** | .34*** | .34*** | .82*** | .36*** | .31*** | .33*** | .20*** | .25*** | .28*** | .35*** | .29*** | .28*** | .32*** | .38*** | — |  |  |  |
| 24 | NC | .48*** | .43*** | .15*** | .64*** | .69*** | .81*** | .35*** | .27*** | .40*** | .34*** | .39*** | .89*** | .41*** | .39*** | .19*** | .26*** | .21*** | .40*** | .34*** | .30*** | .29*** | .36*** | .30*** | — |  |  |
| 25 | NO | .52*** | .49*** | .28*** | .68*** | .63*** | .55*** | .36*** | .33*** | .45*** | .35*** | .41*** | .89*** | .45*** | .41*** | .19*** | .29*** | .29*** | .44*** | .40*** | .28*** | .33*** | .35*** | .33*** | .57*** | — |  |
| 26 | SR | .43*** | .39*** | .23*** | .61*** | .64*** | .46*** | .31*** | .29*** | .36*** | .26*** | .37*** | .54*** | .82*** | .31*** | .20*** | .29*** | .22*** | .34*** | .33*** | .22*** | .24*** | .33*** | .29*** | .44*** | .53*** | — |
| 27 | PR | .23*** | .13*** | .08* | .41*** | .30*** | .24*** | .19*** | .15*** | .22*** | .21*** | .24*** | .24*** | .79*** | .15*** | .17*** | .15*** | .12*** | .19*** | .22*** | .16*** | .20*** | .18*** | .22*** | .23*** | .20*** | .30*** |

*Note.* Sex: -1 = male, 1 = female; SES = socioeconomic status: -1 = no postsecondary maternal education, 1 = postsecondary maternal education; BML = bi/multilingualism: -1 = yes, 1 = no. RLA = receptive language ability; ELA = expressive language ability; PrIQ = Profile IQ; FSIQ = Full-Scale IQ; ScrIQ = Screening IQ; VP = Visual Processing; PS = Processing Speed; ASTM = Auditory Short-Term Memory; VSTM = Visuospatial Short-Term Memory; AR = Abstract Reasoning; VR = Verbal Reasoning; LTM = Long-Term Memory; SD = Shape Design; WD = Washer Design; PSP = Parrots; PSB = Boxes; DLS = Digit and Letter Span; MDLS = Mixed Digit and Letter Span; SM = Shape Memory; RSM = Rotated Shape Memory; MC = Matrices: Completion; MOO = Matrices: Odd One Out; NC = Naming Categories; NO = Naming Opposites; SR = Story Recall; PR = Picture Recall.

**p* < .05. ***p* < .01. ****p* < .001.

**Table S4**

*Post-Hoc Pearson Correlations of the Language Aspects and Scores of the Executive Functions Domain From the Intelligence and Development Scales–2*

| Variable | | 1 | 2 | 3 | 4 | 5 | 6 | 7 |
| --- | --- | --- | --- | --- | --- | --- | --- | --- |
| 1 | RLA | — |  |  |  |  |  |  |
| 2 | ELA | .45*** | — |  |  |  |  |  |
| 3 | BML | .20*** | .23*** | — |  |  |  |  |
| 4 | EFC | .44*** | .37*** | .10** | — |  |  |  |
| 5 | LW | .40*** | .32*** | .12*** | .77*** | — |  |  |
| 6 | DA | .38*** | .33*** | .15*** | .79*** | .59*** | — |  |
| 7 | AC | .25*** | .19*** | -.02 | .68*** | .29*** | .40*** | — |
| 8 | DR | .19*** | .19*** | .04 | .53*** | .21*** | .23*** | .10** |

*Note.* Sex: -1 = male, 1 = female; SES = socioeconomic status: -1 = no postsecondary maternal education, 1 = postsecondary maternal education; BML = bi/multilingualism: -1 = yes, 1 = no. RLA = receptive language ability; ELA = expressive language ability; EFC = Executive functions composite; LW = Listing Words; DA = Divided Attention; AC = Animal Colors; DR = Drawing Routes.

***p* < .01. ****p* < .001.

**Table S5**

*Post-Hoc Pearson Correlations of the Language Aspects and Scores of the Psychomotor Skills Domain From the Intelligence and Development Scales–2*

| Variable | | 1 | 2 | 3 | 4 | 5 | 6 |
| --- | --- | --- | --- | --- | --- | --- | --- |
| 1 | RLA | — |  |  |  |  |  |
| 2 | ELA | .45*** | — |  |  |  |  |
| 3 | BML | .20*** | .23*** | — |  |  |  |
| 4 | PSC | .35*** | .28*** | .05 | — |  |  |
| 5 | GM | .23*** | .19*** | .03 | .79*** | — |  |
| 6 | FM | .25*** | .19*** | .06 | .72*** | .31*** | — |
| 7 | VM | .30*** | .25*** | .01 | .64*** | .24*** | .29*** |

*Note.* Sex: -1 = male, 1 = female; SES = socioeconomic status: -1 = no postsecondary maternal education, 1 = postsecondary maternal education; BML = bi/multilingualism: -1 = yes, 1 = no. RLA = receptive language ability; ELA = expressive language ability; PSC = Psychomotor skills composite; GM = Gross Motor Skills; FM = Fine Motor Skills; VM = Visuomotor Skills.

****p* < .001.

**Table S6**

*Post-Hoc Pearson Correlations of the Language Aspects and Scores of the Social-Emotional Skills Domain From the Intelligence and Development Scales–2*

| Variable | | 1 | 2 | 3 | 4 | 5 | 6 |
| --- | --- | --- | --- | --- | --- | --- | --- |
| 1 | RLA | — |  |  |  |  |  |
| 2 | ELA | .45*** | — |  |  |  |  |
| 3 | BML | .20*** | .23*** | — |  |  |  |
| 4 | SESC | .22*** | .24*** | .05 | — |  |  |
| 5 | IE | .21*** | .20*** | .05 | .67*** | — |  |
| 6 | RE | .18*** | .21*** | .06 | .75*** | .28*** | — |
| 7 | SC | .11** | .13*** | -.01 | .76*** | .25*** | .33*** |

*Note.* Sex: -1 = male, 1 = female; SES = socioeconomic status: -1 = no postsecondary maternal education, 1 = postsecondary maternal education; BML = bi/multilingualism: -1 = yes, 1 = no. RLA = receptive language ability; ELA = expressive language ability; SESC = Social-emotional skills composite; IE = Identifying Emotions; RE = Regulating Emotions; SC = Socially Competent Behavior.

***p* < .01. ****p* < .001.

**Table S7**

*Post-Hoc Pearson Correlations of the Language Aspects and Scores of the Basic Skills Domain From the Intelligence and Development Scales–2*

| Variable | | 1 | 2 | 3 | 4 | 5 |
| --- | --- | --- | --- | --- | --- | --- |
| 1 | RLA | — |  |  |  |  |
| 2 | ELA | .45*** | — |  |  |  |
| 3 | BML | .20*** | .23*** | — |  |  |
| 4 | MR | .47*** | .45*** | .08* | — |  |
| 5 | RD | .41*** | .43*** | .09* | .47*** | — |
| 6 | SP | .39*** | .42*** | .15*** | .45*** | .64*** |

*Note.* Sex: -1 = male, 1 = female; SES = socioeconomic status: -1 = no postsecondary maternal education, 1 = postsecondary maternal education; BML = bi/multilingualism: -1 = yes, 1 = no. RLA = receptive language ability; ELA = expressive language ability; MR = Logical-Mathematical Reasoning; RD = Reading; SP = Spelling.

**p* < .05. ****p* < .001.

**Table S8**

*Hierarchical Regression Analyses for Sex, SES, Receptive Language Ability, Expressive Language Ability, and Bi/Multilingualism as Predictors of Scores on the Profile IQ, Full-Scale IQ, and Screening IQ From the Intelligence and Development Scales–2*

| Step | Predictor | Profile IQ | | Full-Scale IQ | | Screening IQ | |
| --- | --- | --- | --- | --- | --- | --- | --- |
|  |  | β | *p*_H_ | β | *p*_H_ | β | *p*_H_ |
| 1 | Sex  SES | .04  .25 | .999  **< .001** | -.01  .26 | .999  **< .001** | .03  .21 | .999  **< .001** |
|  | *R*^2^ | .06 | | .07 | | .05 | |
| 2 | Sex  SES  RLA | -.03  .16  .57 | .999  **< .001**  **< .001** | -.08  .17  .56 | .955  **< .001**  **< .001** | -.03  .13  .49 | .999  **.005**  **< .001** |
|  | *R*^2^  Δ*R*^2^ | .38  **.32***** | | .37  **.30***** | | .28  **.23***** | |
| 3 | Sex  SES  RLA  ELA | -.04  .13  .45  .28 | .999  **< .001**  **< .001**  **< .001** | -.09  .14  .44  .28 | .147  **< .001**  **< .001**  **< .001** | -.04  .11  .37  .28 | .999  .086  **< .001**  **< .001** |
|  | *R*^2^  Δ*R*^2^ | .44  **.06***** | | .43  **.06***** | | .34  **.06***** | |
| 4 | Sex  SES  RLA  ELA  BML | -.04  .13  .45  .28  .02 | .999  **< .001**  **< .001**  **< .001**  .999 | -.10  .14  .44  .27  .02 | .143  **< .001**  **< .001**  **< .001**  .999 | -.04  .11  .37  .28  .00 | .999  .088  **< .001**  **< .001**  .999 |
|  | *R*^2^  Δ*R*^2^ | .44  .00 | | .43  .00 | | .34  .00 | |

*Note.* Sex: -1 = male, 1 = female; SES = socioeconomic status: -1 = no postsecondary maternal education, 1 = postsecondary maternal education; BML = bi/multilingualism: -1 = yes, 1 = no; RLA = receptive language ability; ELA = expressive language ability; *p*_H_ = *p* value adjusted with the correction by Hommel (1988). Significant results are presented in bold.

****p*_H_ < .001.

**Table S9**

*Hierarchical Regression Analyses for Sex, SES, Receptive Language Ability, Expressive Language Ability, and Bi/Multilingualism as Predictors of Scores on the Seven Group Factors of the Intelligence Domain From the Intelligence and Development Scales–2*

| Step | Predictor | Visual Processing | | Processing Speed | | Auditory Short-Term Memory | | Visuospatial Short-Term Memory | | Abstract Reasoning | | Verbal Reasoning | | Long-Term Memory | |
| --- | --- | --- | --- | --- | --- | --- | --- | --- | --- | --- | --- | --- | --- | --- | --- |
|  |  | β | *p*_H_ | β | *p*_H_ | β | *p*_H_ | β | *p*_H_ | β | *p*_H_ | β | *p*_H_ | β | *p*_H_ |
| 1 | Sex  SES | -.13  .19 | .058  **< .001** | .05  .12 | .999  .210 | .05  .21 | .999  **< .001** | .03  .13 | .999  .071 | .08  .20 | .999  **< .001** | .04  .23 | .999  **< .001** | .07  .12 | .999  .125 |
|  | *R*^2^ | .05 | | .02 | | .05 | | .02 | | .05 | | .06 | | .02 | |
| 2 | Sex  SES  RLA | -.17  .14  .35 | **< .001**  **.011**  **< .001** | .01  .07  .32 | .999  .999  **< .001** | -.00  .15  .40 | .999  **.002**  **< .001** | -.01  .08  .33 | .999  .999  **< .001** | .03  .14  .39 | .999  **.006**  **< .001** | -.02  .15  .55 | .999  **< .001**  **< .001** | .02  .06  .40 | .999  .999  **< .001** |
|  | *R*^2^  Δ*R*^2^ | .17  **.12***** | | .12  **.10***** | | .20  **.15***** | | .12  **.10***** | | .19  **.14***** | | .34  **.28***** | | .17  **.15***** | |
| 3 | Sex  SES  RLA  ELA | -.18  .12  .27  .17 | **< .001**  .062  **< .001**  **.001** | .00  .06  .27  .11 | .999  .999  **< .001**  .665 | -.01  .13  .30  .23 | .999  **.022**  **< .001**  **< .001** | -.01  .07  .27  .13 | .999  .999  **< .001**  .111 | .02  .12  .30  .20 | .999  **.048**  **< .001**  **< .001** | -.04  .12  .41  .32 | .999  **.011**  **< .001**  **< .001** | .01  .05  .32  .17 | .999  .999  **< .001**  **.002** |
|  | *R*^2^  Δ*R*^2^ | .19  **.02**** | | .13  .01 | | .24  **.04***** | | .13  .01 | | .22  **.03***** | | .42  **.08***** | | .19  **.02**** | |
| 4 | Sex  SES  RLA  ELA  BML | -.18  .12  .28  .18  -.03 | **< .001**  .091  **< .001**  **< .001**  .999 | .00  .05  .28  .12  -.04 | .999  .999  **< .001**  .462  .999 | -.01  .13  .30  .23  .00 | .999  **.023**  **< .001**  **< .001**  .999 | -.01  .06  .27  .14  -.01 | .999  .999  **< .001**  .105  .999 | .02  .12  .31  .21  -.03 | .999  .074  **< .001**  **< .001**  .999 | -.04  .13  .40  .30  .10 | .999  **.002**  **< .001**  **< .001**  .059 | .01  .06  .31  .15  .11 | .999  .999  **< .001**  **.017**  .379 |
|  | *R*^2^  Δ*R*^2^ | .19  .00 | | .13  .00 | | .24  .00 | | .13  .00 | | .23  .01 | | .43  .01 | | .20  .01 | |

*Note.* Sex: -1 = male, 1 = female; SES = socioeconomic status: -1 = no postsecondary maternal education, 1 = postsecondary maternal education; BML = bi/multilingualism: -1 = yes, 1 = no; RLA = receptive language ability; ELA = expressive language ability. *p*_H_ = *p* value adjusted with the correction by Hommel (1988). Significant results are presented in bold.

***p*_H_ < .01. ****p*_H_ < .001.

**Table S10**

*Hierarchical Regression Analyses for Sex, SES, Receptive Language Ability, Expressive Language Ability, and Bi/Multilingualism as Predictors of Scores on the Seven Subtests of the Intelligence Domain (Part 1) From the Intelligence and Development Scales–2*

| Step | Predictor | Shape Design | | Parrots | | Digit and Letter Span | | Shape Memory | | Matrices: Completion | | Naming Categories | | Story Recall | | |
| --- | --- | --- | --- | --- | --- | --- | --- | --- | --- | --- | --- | --- | --- | --- | --- | --- |
|  |  | β | *p*_H_ | β | *p*_H_ | β | *p*_H_ | β | *p*_H_ | β | *p*_H_ | β | *p*_H_ | β | *p*_H_ |  |
| 1 | Sex  SES | -.18  .21 | **< .001**  **< .001** | .05  .12 | .999  .196 | .05  .23 | .999  **< .001** | .02  .12 | .999  .227 | .02  .17 | .999  **< .001** | .03  .18 | .999  **< .001** | -.02  .15 | .999  **.007** |  |
|  | *R*^2^ | .07 | | .02 | | .06 | | .01 | | .03 | | .03 | | .02 | | |
| 2 | Sex  SES  RLA | -.23  .15  .38 | **< .001**  **.002**  **< .001** | .02  .07  .30 | .999  .999  **< .001** | .00  .17  .38 | .999  **< .001**  **< .001** | -.01  .07  .27 | .999  .999  **< .001** | -.02  .11  .35 | .999  .228  **< .001** | -.03  .11  .47 | .999  .223  **< .001** | -.07  .08  .42 | .999  .999  **< .001** |  |
|  | *R*^2^  Δ*R*^2^ | .21  **.14***** | | .10  **.08***** | | .20  **.14***** | | .08  **.07***** | | .14  **.11***** | | .24  **.21***** | | .20  **.18***** | | |
| 3 | Sex  SES  RLA  ELA | -.24  .13  .32  .14 | **< .001**  **.011**  **< .001**  **.024** | .01  .06  .24  .13 | .999  .999  **< .001**  .141 | -.01  .15  .29  .20 | .999  **.002**  **< .001**  **< .001** | -.01  .07  .23  .09 | .999  .999  **< .001**  .999 | -.03  .09  .26  .21 | .999  .957  **< .001**  **< .001** | -.04  .08  .36  .26 | .999  .993  **< .001**  **< .001** | -.09  .06  .32  .25 | .961  .999  **< .001**  **< .001** |  |
|  | *R*^2^  Δ*R*^2^ | .23  **.02*** | | .11  .01 | | .23  **.03***** | | .09  .01 | | .18  **.04***** | | .30  **.06***** | | .24  **.04***** | | |
| 4 | Sex  SES  RLA  ELA  BML | -.24  .13  .33  .15  -.05 | **< .001**  **.021**  **< .001**  **.010**  .999 | .01  .06  .24  .13  -.01 | .999  .999  **< .001**  .137  .999 | -.01  .15  .29  .20  .01 | .999  **.001**  **< .001**  **< .001**  .999 | -.01  .07  .23  .09  -.01 | .999  .999  **< .001**  .999  .999 | -.03  .09  .26  .21  -.02 | .999  .990  **< .001**  **< .001**  .999 | -.04  .08  .35  .25  .03 | .999  .990  **< .001**  **< .001**  .999 | -.09  .07  .30  .23  .13 | .787  .999  **< .001**  **< .001**  **.022** |  |
|  | *R*^2^  Δ*R*^2^ | .23  .00 | | .11  .00 | | .23  .00 | | .09  .00 | | .18  .00 | | .30  .00 | | .26  **.02*** | | |

*Note.* Sex: -1 = male, 1 = female; SES = socioeconomic status: -1 = no postsecondary maternal education, 1 = postsecondary maternal education; BML = bi/multilingualism: -1 = yes, 1 = no; RLA = receptive language ability; ELA = expressive language ability; *p*_H_ = *p* value adjusted with the correction by Hommel (1988). Significant results are presented in bold.

**p*_H_ < .05. ****p*_H_ < .001.

**Table S11**

*Hierarchical Regression Analyses for Sex, SES, Receptive Language Ability, Expressive Language Ability, and Bi/Multilingualism as Predictors of Scores on the Seven Subtests of the Intelligence Domain (Part 2) From the Intelligence and Development Scales–2*

| Step | Predictor | Washer Design | | Boxes | | Mixed Digit and Letter Span | | Rotated Shape Memory | | Matrices: Odd One Out | | Naming Opposites | | Picture Recall | | |
| --- | --- | --- | --- | --- | --- | --- | --- | --- | --- | --- | --- | --- | --- | --- | --- | --- |
|  |  | β | *p*_H_ | β | *p*_H_ | β | *p*_H_ | β | *p*_H_ | β | *p*_H_ | β | *p*_H_ | β | *p*_H_ |  |
| 1 | Sex  SES | -.03  .11 | .999  .555 | .03  .10 | .999  .987 | .04  .17 | .999  **.001** | .04  .10 | .999  .915 | .12  .17 | .219  **< .001** | .05  .23 | .999  **< .001** | .14  .05 | **.034**  .999 |  |
|  | *R*^2^ | .01 | | .01 | | .03 | | .01 | | .04 | | .06 | | .02 | | |
| 2 | Sex  SES  RLA | -.05  .08  .20 | .999  .999  **< .001** | -.00  .06  .26 | .999  .999  **< .001** | -.00  .11  .37 | .999  .336  **< .001** | .00  .05  .30 | .999  .999  **< .001** | .08  .12  .30 | .999  .092  **< .001** | -.01  .15  .50 | .999  **< .001**  **< .001** | .11  .02  .21 | .376  .999  **< .001** |  |
|  | *R*^2^  Δ*R*^2^ | .05  **.04***** | | .08  **.07***** | | .16  **.13***** | | .10  **.09***** | | .13  **.09***** | | .30  **.24***** | | .06  **.04***** | | |
| 3 | Sex  SES  RLA  ELA | -.06  .06  .13  .14 | .999  .999  .165  .102 | -.01  .05  .24  .06 | .999  .999  **< .001**  .999 | -.01  .09  .28  .21 | .999  .993  **< .001**  **< .001** | -.00  .04  .23  .16 | .999  .999  **< .001**  **.006** | .07  .11  .25  .12 | .999  .296  **< .001**  .268 | -.03  .12  .37  .31 | .999  **.013**  **< .001**  **< .001** | .11  .01  .20  .02 | .420  .999  **< .001**  .999 |  |
|  | *R*^2^  Δ*R*^2^ | .06  .01 | | .08  .00 | | .20  **.04***** | | .12  **.02**** | | .14  .01 | | .37  **.07***** | | .07  .01 | | |
| 4 | Sex  SES  RLA  ELA  BML | -.06  .06  .14  .14  -.00 | .999  .999  .174  .118  .999 | -.01  .05  .24  .07  -.05 | .999  .999  **< .001**  .999  .999 | -.01  .09  .28  .21  -.02 | .999  .996  **< .001**  **< .001**  .999 | -.00  .03  .24  .17  -.03 | .999  .999  **< .001**  **.005**  .999 | .08  .11  .25  .13  -.04 | .999  .428  **< .001**  .172  .999 | -.03  .13  .35  .28  .16 | .999  **.001**  **< .001**  **< .001**  **< .001** | .11  .02  .20  .01  .03 | .444  .999  **< .001**  .999  .999 |  |
|  | *R*^2^  Δ*R*^2^ | .06  .00 | | .08  .00 | | .20  .00 | | .12  .00 | | .14  .00 | | .39  **.02***** | | .07  .01 | | |

*Note.* Sex: -1 = male, 1 = female; SES = socioeconomic status: -1 = no postsecondary maternal education, 1 = postsecondary maternal education; BML = bi/multilingualism: -1 = yes, 1 = no; RLA = receptive language ability; ELA = expressive language ability; *p*_H_ = *p* value adjusted with the correction by Hommel (1988). Significant results are presented in bold.

***p*_H_ < .01. ****p*_H_ < .001.

**Table S12**

*Hierarchical Regression Analyses for Sex, SES, Receptive Language Ability, Expressive Language Ability, and Bi/Multilingualism as Predictors of Scores on the Composite and Subtests of the Executive Functions Domain From the Intelligence and Development Scales–2*

| Step | Predictor | Executive functions composite | | Listing Words | | Divided Attention | | Animal Colors | | Drawing Routes | | |
| --- | --- | --- | --- | --- | --- | --- | --- | --- | --- | --- | --- | --- |
|  |  | β | *p*_H_ | β | *p*_H_ | β | *p*_H_ | β | *p*_H_ | β | *p*_H_ |  |
| 1 | Sex  SES | .09  .17 | .998  **< .001** | .14  .16 | **.029**  **.003** | .05  .13 | .999  .106 | .09  .06 | .999  .999 | -.05  .13 | .999  .103 |  |
|  | *R*^2^ | .04 | | .04 | | .02 | | .01 | | .02 | | |
| 2 | Sex  SES  RLA | .04  .11  .42 | .999  .302  **< .001** | .09  .10  .38 | .993  .549  **< .001** | .01  .07  .37 | .999  .999  **< .001** | .06  .02  .23 | .999  .999  **< .001** | -.08  .10  .18 | .999  .959  **< .001** |  |
|  | *R*^2^  Δ*R*^2^ | .21  **.17***** | | .18  **.14***** | | .15  **.13***** | | .06  **.05***** | | .05  **.03***** | | |
| 3 | Sex  SES  RLA  ELA | .03  .09  .33  .20 | .999  .990  **< .001**  **< .001** | .08  .09  .31  .16 | .999  .995  **< .001**  **.007** | .00  .06  .29  .19 | .999  .999  **< .001**  **< .001** | .05  .02  .19  .10 | .999  .999  **< .001**  .999 | -.08  .09  .13  .13 | .999  .999  .347  .389 |  |
|  | *R*^2^  Δ*R*^2^ | .24  **.03***** | | .20  **.02**** | | .18  **.03***** | | .07  .01 | | .06  .01 | | |
| 4 | Sex  SES  RLA  ELA  BML | .03  .09  .33  .21  -.02 | .999  .993  **< .001**  **< .001**  .999 | .08  .09  .31  .15  .02 | .999  .993  **< .001**  **.015**  .999 | .00  .06  .28  .18  .05 | .999  .999  **< .001**  **< .001**  .999 | .06  .01  .21  .11  -.09 | .999  .999  **< .001**  .946  .998 | -.08  .09  .13  .13  -.01 | .999  .999  .354  .408  .999 |  |
|  | *R*^2^  Δ*R*^2^ | .24  .00 | | .20  .00 | | .18  .00 | | .08  .01 | | .06  .00 | | |

*Note.* Sex: -1 = male, 1 = female; SES = socioeconomic status: -1 = no postsecondary maternal education, 1 = postsecondary maternal education; BML = bi/multilingualism: -1 = yes, 1 = no; RLA = receptive language ability; ELA = expressive language ability; *p*_H_ = *p* value adjusted with the correction by Hommel (1988). Significant results are presented in bold.

***p*_H_ < .01. ****p*_H_ < .001.

**Table S13**

*Hierarchical Regression Analyses for Sex, SES, Receptive Language Ability, Expressive Language Ability, and Bi/Multilingualism as Predictors of Scores on the Composite and Subtests of the Psychomotor Skills Domain From the Intelligence and Development Scales–2*

| Step | Predictor | Psychomotor skills composite | | Gross Motor Skills | | Fine Motor Skills | | Visuomotor Skills | |
| --- | --- | --- | --- | --- | --- | --- | --- | --- | --- |
|  |  | β | *p*_H_ | β | *p*_H_ | β | *p*_H_ | β | *p*_H_ |
| 1 | Sex  SES | .11  .08 | .268  .999 | .00  .05 | .999  .999 | .18  .02 | **< .001**  .999 | .09  .12 | .993  .277 |
|  | *R*^2^ | .02 | | .00 | | .03 | | .02 | |
| 2 | Sex  SES  RLA | .08  .02  .33 | .999  .999  **< .001** | -.02  .01  .23 | .999  .999  **< .001** | .15  -.02  .23 | **.002**  .999  **< .001** | .06  .07  .28 | .999  .999  **< .001** |
|  | *R*^2^  Δ*R*^2^ | .13  **.11***** | | .05  **.05***** | | .09  **.06***** | | .10  **.08***** | |
| 3 | Sex  SES  RLA  ELA | .07  .01  .27  .15 | .999  .999  **< .001**  **.010** | -.03  -.00  .18  .11 | .999  .999  **.001**  .982 | .15  -.03  .19  .09 | **.005**  .999  **< .001**  .999 | .06  .06  .22  .13 | .999  .999  **< .001**  .110 |
|  | *R*^2^  Δ*R*^2^ | .15  **.02**** | | .06  .01 | | .09  .00 | | .11  .01 | |
| 4 | Sex  SES  RLA  ELA  BML | .07  .01  .27  .16  -.05 | .999  .999  **< .001**  **.005**  .999 | -.03  -.00  .19  .11  -.03 | .999  .999  **< .001**  .801  .999 | .15  -.03  .19  .10  -.01 | **.005**  .999  **< .001**  .999  .999 | .06  .05  .23  .15  -.07 | .999  .999  **< .001**  **.039**  .999 |
|  | *R*^2^  Δ*R*^2^ | .15  .00 | | .06  .00 | | .09  .00 | | .12  .01 | |

*Note.* Sex: -1 = male, 1 = female; SES = socioeconomic status: -1 = no postsecondary maternal education, 1 = postsecondary maternal education; BML = bi/multilingualism: -1 = yes, 1 = no; RLA = receptive language ability; ELA = expressive language ability; *p*_H_ = *p* value adjusted with the correction by Hommel (1988). Significant results are presented in bold.

***p*_H_ < .01. ****p*_H_ < .001.

**Table S14**

*Hierarchical Regression Analyses for Sex, SES, Receptive Language Ability, Expressive Language Ability, and Bi/Multilingualism as Predictors of Scores on the Composite and Subtests of the Social-Emotional Skills Domain From the Intelligence and Development Scales–2*

| Step | Predictor | Social-emotional skills composite | | Identifying Emotions | | Regulating Emotions | | Socially Competent Behavior | |
| --- | --- | --- | --- | --- | --- | --- | --- | --- | --- |
|  |  | β | *p*_H_ | β | *p*_H_ | β | *p*_H_ | β | *p*_H_ |
| 1 | Sex  SES | .07  .07 | .999  .999 | .05  .09 | .999  .999 | .04  .03 | .999  .999 | .05  .03 | .999  .999 |
|  | *R*^2^ | .01 | | .01 | | .00 | | .00 | |
| 2 | Sex  SES  RLA | .04  .03  .21 | .999  .999  **< .001** | .03  .06  .19 | .999  .999  **< .001** | .02  -.00  .17 | .999  .999  **< .001** | .04  .02  .10 | .999  .999  .742 |
|  | *R*^2^  Δ*R*^2^ | .05  **.04***** | | .05  **.04***** | | .03  **.03***** | | .01  .01 | |
| 3 | Sex  SES  RLA  ELA | .03  .02  .14  .18 | .999  .999  .105  **.002** | .02  .05  .14  .12 | .999  .999  .081  .471 | .01  -.02  .10  .17 | .999  .999  .996  **.006** | .03  .01  .06  .10 | .999  .999  .999  .999 |
|  | *R*^2^  Δ*R*^2^ | .08  **.03**** | | .06  .01 | | .05  **.02**** | | .02  .01 | |
| 4 | Sex  SES  RLA  ELA  BML | .03  .01  .14  .18  -.02 | .999  .999  .086  **.001**  .999 | .02  .05  .14  .12  -.01 | .999  .999  .085  .502  .999 | .01  -.02  .10  .17  -.00 | .999  .999  .997  **.007**  .999 | .03  .00  .07  .11  -.05 | .999  .999  .999  .993  .999 |
|  | *R*^2^  Δ*R*^2^ | .08  .00 | | .06  .00 | | .05  .00 | | .02  .00 | |

*Note.* Sex: -1 = male, 1 = female; SES = socioeconomic status: -1 = no postsecondary maternal education, 1 = postsecondary maternal education; BML = bi/multilingualism: -1 = yes, 1 = no; RLA = receptive language ability; ELA = expressive language ability; *p*_H_ = *p* value adjusted with the correction by Hommel (1988). Significant results are presented in bold.

***p*_H_ < .01. ****p*_H_ < .001.

**Table S15**

*Hierarchical Regression Analyses for Sex, SES, Receptive Language Ability, Expressive Language Ability, and Bi/Multilingualism as Predictors of Scores on the Subtests of the Basic Skills Domain From the Intelligence and Development Scales–2*

| Step | Predictor | Logical-Mathematical Reasoning | | Reading | | Spelling | |
| --- | --- | --- | --- | --- | --- | --- | --- |
|  |  | β | *p*_H_ | β | *p*_H_ | β | *p*_H_ |
| 1 | Sex  SES | -.07  .22 | .999  **< .001** | .05  .15 | .999  .091 | .21  .06 | **< .001**  .999 |
|  | *R*^2^ | .05 | | .03 | | .05 | |
| 2 | Sex  SES  RLA | -.12  .14  .46 | **.021**  **.001**  **< .001** | -.01  .09  .39 | .999  .999  **< .001** | .16  .01  .36 | **.040**  .999  **< .001** |
|  | *R*^2^  Δ*R*^2^ | .26  **.21***** | | .17  **.14***** | | .17  **.12***** | |
| 3 | Sex  SES  RLA  ELA | -.14  .12  .34  .29 | **< .001**  **.028**  **< .001**  **< .001** | -.02  .06  .26  .29 | .999  .999  **< .001**  **< .001** | .14  -.02  .23  .31 | .094  .999  **< .001**  **< .001** |
|  | *R*^2^  Δ*R*^2^ | .32  **.06***** | | .24  **.07***** | | .25  **.08***** | |
| 4 | Sex  SES  RLA  ELA  BML | -.14  .11  .35  .30  -.05 | **< .001**  .052  **< .001**  **< .001**  .999 | -.02  .05  .27  .30  -.03 | .999  .999  **< .001**  **< .001**  .999 | .14  -.02  .23  .30  .02 | .095  .999  **< .001**  **< .001**  .999 |
|  | *R*^2^  Δ*R*^2^ | .33  .01 | | .24  .00 | | .25  .00 | |

*Note.* Sex: -1 = male, 1 = female; SES = socioeconomic status: -1 = no postsecondary maternal education, 1 = postsecondary maternal education; BML = bi/multilingualism: -1 = yes, 1 = no; RLA = receptive language ability; ELA = expressive language ability; *p*_H_ = *p* value adjusted with the correction by Hommel (1988). The basic skills composite was not investigated since the receptive and expressive language abilities are also included in the calculation of this score. Significant results are presented in bold.

****p*_H_ < .001.

**Table S16**

*Hierarchical Regression Analyses for Sex, SES, Intelligence, Receptive Language Ability, and Expressive Language Ability as Predictors of Scores on the Composite and Subtests of the Executive Functions Domain From the Intelligence and Development Scales–2*

| Step | Predictor | Executive functions composite | | Listing Words | | Divided Attention | | Animal Colors | | Drawing Routes | | |
| --- | --- | --- | --- | --- | --- | --- | --- | --- | --- | --- | --- | --- |
|  |  | β | *p*_H_ | β | *p*_H_ | β | *p*_H_ | β | *p*_H_ | β | *p*_H_ |  |
| 1 | Sex  SES | .09  .16 | .938  **.001** | .14  .15 | **.017**  **.004** | .05  .12 | .974  .117 | .09  .05 | .956  .974 | -.06  .12 | .974  .133 |  |
|  | *R*^2^ | .04 | | .04 | | .02 | | .01 | | .02 | | |
| 2 | Sex  SES  IQ | .07  .01  .64 | .972  .974  **< .001** | .12  .03  .52 | **.017**  .974  **< .001** | .04  -.02  .57 | .974  .974  **< .001** | .08  -.04  .36 | .974  .974  **< .001** | -.07  .04  .33 | .974  .974  **< .001** |  |
|  | *R*^2^  Δ*R*^2^ | .42  **.38***** | | .29  **.25***** | | .32  **.30***** | | .14  **.13***** | | .12  **.10***** | | |
| 3 | Sex  SES  IQ  RLA | .06  .01  .58  .10 | .974  .974  **< .001**  .559 | .11  .03  .44  .12 | .093  .974  **< .001**  .159 | .03  -.02  .53  .08 | .974  .974  **< .001**  .974 | .07  -.04  .34  .05 | .974  .974  **< .001**  .974 | -.07  .04  .33  .00 | .974  .974  **< .001**  .974 |  |
|  | *R*^2^  Δ*R*^2^ | .42  .00 | | .30  .01 | | .33  .01 | | .14  .00 | | .12  .00 | | |
| 4 | Sex  SES  IQ  RLA  ELA | .05  .00  .56  .09  .05 | .974  .974  **< .001**  .958  .974 | .10  .02  .43  .12  .03 | .120  .974  **< .001**  .349  .974 | .02  -.02  .51  .07  .05 | .974  .974  **< .001**  .974  .974 | .07  -.04  .33  .05  .01 | .974  .974  **< .001**  .974  .974 | -.07  .04  .32  -.01  .03 | .974  .974  **< .001**  .974  .974 |  |
|  | *R*^2^  Δ*R*^2^ | .42  .00 | | .31  .01 | | .33  .00 | | .14  .00 | | .12  .00 | | |

*Note.* Sex: -1 = male, 1 = female; SES = socioeconomic status: -1 = no postsecondary maternal education, 1 = postsecondary maternal education; IQ = intelligence (Profile IQ); RLA = receptive language ability; ELA = expressive language ability; *p*_H_ = *p* value adjusted with the correction by Hommel (1988). As bi/multilingualism did not explain additional variance in scores in the executive functions domain in the previous hierarchical regression analyses (see Table S12), this variable was omitted from the present analyses. Significant results are presented in bold.

****p*_H_ < .001.

**Table S17**

*Hierarchical Regression Analyses for Sex, SES, Intelligence, Receptive Language Ability, and Expressive Language Ability as Predictors of Scores on the Composite and Subtests of the Psychomotor Skills Domain From the Intelligence and Development Scales–2*

| Step | Predictor | Psychomotor skills composite | | Gross Motor Skills | | Fine Motor Skills | | Visuomotor Skills | |
| --- | --- | --- | --- | --- | --- | --- | --- | --- | --- |
|  |  | β | *p*_H_ | β | *p*_H_ | β | *p*_H_ | β | *p*_H_ |
| 1 | Sex  SES | .12  .07 | .095  .974 | .01  .04 | .974  .974 | .18  .01 | **< .001**  .974 | .10  .11 | .814  .412 |
|  | *R*^2^ | .02 | | .00 | | .03 | | .02 | |
| 2 | Sex  SES  IQ | .10  -.06  .52 | .138  .974  **< .001** | .00  -.04  .33 | .974  .974  **< .001** | .17  -.08  .36 | **< .001**  .974  **< .001** | .08  -.01  .48 | .850  .974  **< .001** |
|  | *R*^2^  Δ*R*^2^ | .27  **.25***** | | .10  **.10***** | | .15  **.12***** | | .24  **.22***** | |
| 3 | Sex  SES  IQ  RLA | .10  -.06  .48  .07 | .285  .974  **< .001**  .974 | -.00  -.04  .29  .07 | .974  .974  **< .001**  .974 | .17  -.08  .33  .05 | **< .001**  .974  **< .001**  .974 | .08  -.01  .46  .03 | .946  .974  **< .001**  .974 |
|  | *R*^2^  Δ*R*^2^ | .27  .00 | | .11  .01 | | .16  .01 | | .24  .00 | |
| 4 | Sex  SES  IQ  RLA  ELA | .09  -.06  .47  .06  .02 | .329  .974  **< .001**  .974  .974 | -.01  -.04  .28  .06  .03 | .974  .974  **< .001**  .974  .974 | .17  -.08  .33  .05  .01 | **< .001**  .974  **< .001**  .974  .974 | .08  -.01  .46  .02  .01 | .956  .974  **< .001**  .974  .974 |
|  | *R*^2^  Δ*R*^2^ | .27  .00 | | .11  .00 | | .16  .00 | | .24  .00 | |

*Note.* Sex: -1 = male, 1 = female; SES = socioeconomic status: -1 = no postsecondary maternal education, 1 = postsecondary maternal education; IQ = intelligence (Profile IQ); RLA = receptive language ability; ELA = expressive language ability; *p*_H_ = *p* value adjusted with the correction by Hommel (1988). As bi/multilingualism did not explain additional variance in scores of the psychomotor skills domain in the previous hierarchical regression analyses (see Table S13), this variable was omitted from the present analyses. Significant results are presented in bold.

****p*_H_ < .001.

**Table S18**

*Hierarchical Regression Analyses for Sex, SES, Intelligence, Receptive Language Ability, and Expressive Language Ability as Predictors of Scores on the Composite and Subtests of the Social-Emotional Skills Domain From the Intelligence and Development Scales–2*

| Step | Predictor | Social-emotional skills composite | | Identifying Emotions | | Regulating Emotions | | Socially Competent Behavior | |
| --- | --- | --- | --- | --- | --- | --- | --- | --- | --- |
|  |  | β | *p*_H_ | β | *p*_H_ | β | *p*_H_ | β | *p*_H_ |
| 1 | Sex  SES | .06  .07 | .974  .974 | .05  .09 | .974  .963 | .04  .02 | .974  .974 | .04  .03 | .974  .974 |
|  | *R*^2^ | .01 | | .01 | | .00 | | .00 | |
| 2 | Sex  SES  IQ | .05  -.00  .28 | .974  .974  **< .001** | .04  .04  .19 | .974  .974  **< .001** | .03  -.04  .26 | .974  .974  **< .001** | .04  -.00  .15 | .974  .974  **.005** |
|  | *R*^2^  Δ*R*^2^ | .08  **.07***** | | .04  **.03***** | | .07  **.07***** | | .03  **.03**** | |
| 3 | Sex  SES  IQ  RLA | .04  -.01  .22  .10 | .974  .974  **< .001**  .974 | .03  .04  .10  .15 | .974  .974  .974  .132 | .02  -.04  .24  .05 | .974  .974  **< .001**  .974 | .03  -.01  .13  .03 | .974  .974  .426  .974 |
|  | *R*^2^  Δ*R*^2^ | .09  .01 | | .06  .02 | | .07  .00 | | .03  .00 | |
| 4 | Sex  SES  IQ  RLA  ELA | .03  -.01  .17  .07  .13 | .974  .974  **.023**  .974  .167 | .02  .04  .06  .12  .10 | .974  .974  .974  .715  .930 | .01  -.05  .20  .02  .12 | .974  .974  **.004**  .974  .553 | .03  -.01  .11  .02  .07 | .974  .974  .974  .974  .974 |
|  | *R*^2^  Δ*R*^2^ | .10  .01 | | .06  .00 | | .08  .01 | | .03  .00 | |

*Note.* Sex: -1 = male, 1 = female; SES = socioeconomic status: -1 = no postsecondary maternal education, 1 = postsecondary maternal education; IQ = intelligence (Profile IQ); RLA = receptive language ability; ELA = expressive language ability; *p*_H_ = *p* value adjusted with the correction by Hommel (1988). As bi/multilingualism did not explain additional variance in scores of the social-emotional skills domain in the previous hierarchical regression analyses (see Table S14), this variable was omitted from the present analyses. Significant results are presented in bold.

***p*_H_ < .01. ****p*_H_ < .001.

**Table S19**

*Hierarchical Regression Analyses for Sex, SES, Intelligence, Receptive Language Ability, and Expressive Language Ability as Predictors of Scores on the Subtests of the Basic Skills Domain From the Intelligence and Development Scales–2*

| Step | Predictor | Logical-Mathematical Reasoning | | Reading | | Spelling | |
| --- | --- | --- | --- | --- | --- | --- | --- |
|  |  | β | *p*_H_ | β | *p*_H_ | β | *p*_H_ |
| 1 | Sex  SES | -.07  .22 | .974  **< .001** | .05  .15 | .974  .078 | .21  .06 | **< .001**  .974 |
|  | *R*^2^ | .05 | | .03 | | .05 | |
| 2 | Sex  SES  IQ | -.09  .06  .62 | .124  .974  **< .001** | .02  .02  .53 | .974  .974  **< .001** | .18  -.07  .54 | **< .001**  .974  **< .001** |
|  | *R*^2^  Δ*R*^2^ | .41  **.36***** | | .28  **.25***** | | .32  **.27***** | |
| 3 | Sex  SES  IQ  RLA | -.11  .06  .53  .16 | **.013**  .974  **< .001**  **< .001** | .01  .02  .44  .14 | .974  .974  **< .001**  .308 | .17  -.07  .49  .08 | **< .001**  .974  **< .001**  .974 |
|  | *R*^2^  Δ*R*^2^ | .43  **.02***** | | .30  .02 | | .32  .00 | |
| 4 | Sex  SES  IQ  RLA  ELA | -.12  .05  .47  .13  .16 | **.002**  .974  **< .001**  **.034**  **< .001** | -.00  .00  .37  .10  .19 | .974  .974  **< .001**  .974  **.002** | .16  -.08  .42  .04  .20 | **.002**  .974  **< .001**  .974  **.001** |
|  | *R*^2^  Δ*R*^2^ | .45  **.02***** | | .32  **.02**** | | .35  **.03**** | |

*Note.* Sex: -1 = male, 1 = female; SES = socioeconomic status: -1 = no postsecondary maternal education, 1 = postsecondary maternal education; IQ = intelligence (Profile IQ); RLA = receptive language ability; ELA = expressive language ability; *p*_H_ = *p* value adjusted with the correction by Hommel (1988). The basic skills composite was not investigated since the receptive and expressive language abilities are also included in the calculation of this score. As bi/multilingualism did not explain additional variance in scores of the basic skills domain in the previous hierarchical regression analyses (see Table S15), this variable was omitted from the present analyses. Significant results are presented in bold.

***p*_H_ < .01. ****p*_H_ < .001.

**Table S20**

*Post-Hoc Hierarchical Regression Analyses for Sex, SES, Age, Receptive Language Ability, Expressive Language Ability, and Bi/Multilingualism as Predictors and Moderator (Age) of Scores on the Profile IQ, Full-Scale IQ, and Screening IQ From the Intelligence and Development Scales–2*

| Step | Predictor | Profile IQ | | Full-Scale IQ | | | Screening IQ | |
| --- | --- | --- | --- | --- | --- | --- | --- | --- |
|  |  | β | *p*_H_ | β | | *p*_H_ | β | *p*_H_ |
| 1 | Sex | .04 | .999 | -.01 | | .999 | .03 | .999 |
|  | SES | .25 | **< .001** | .26 | | **< .001** | .21 | **< .001** |
|  | Age | -.02 | .999 | -.01 | | .999 | -.00 | .999 |
|  | *R*^2^ | .06 | | .07 | | | .05 | |
| 2 | Sex | -.03 | .999 | -.08 | | .999 | -.03 | .999 |
|  | SES | .16 | **< .001** | .17 | | **< .001** | .13 | **.013** |
|  | Age | -.02 | .999 | -.01 | | .999 | -.00 | .999 |
|  | RLA | .57 | **< .001** | .56 | | **< .001** | .49 | **< .001** |
|  | *R*^2^ | .38 | | .37 | | | .28 | |
|  | Δ*R*^2^ | **.32***** | | **.30***** | | | **.23***** | |
| 3 | Sex | -.04 | .999 | -.09 | | .375 | -.04 | .999 |
|  | SES | .13 | **.001** | .14 | | **< .001** | .11 | .216 |
|  | Age | -.01 | .999 | -.01 | | .999 | .00 | .999 |
|  | RLA | .45 | **< .001** | .44 | | **< .001** | .37 | **< .001** |
|  | ELA | .28 | **< .001** | .28 | | **< .001** | .28 | **< .001** |
|  | *R*^2^ | .44 | | .43 | | | .34 | |
|  | Δ*R*^2^ | **.06***** | | **.06***** | | | **.06***** | |
| 4 | Sex | -.05 | .999 | -.10 | | .364 | -.04 | .999 |
|  | SES | .13 | **.001** | .14 | | **< .001** | .11 | .222 |
|  | Age | -.01 | .999 | -.01 | | .999 | .00 | .999 |
|  | RLA | .45 | **< .001** | .44 | | **< .001** | .37 | **< .001** |
|  | ELA | .28 | **< .001** | .27 | | **< .001** | .28 | **< .001** |
|  | BML | .02 | .999 | .02 | | .999 | .00 | .999 |
|  | *R*^2^ | .44 | | .43 | | | .34 | |
|  | Δ*R*^2^ | .00 | | .00 | | | .00 | |
| 5 | Sex | -.04 | .999 | -.10 | | .375 | -.04 | .999 |
|  | SES | .13 | **.001** | .14 | | **< .001** | .11 | .162 |
|  | Age | -.17 | .999 | -.18 | .999 | | -.40 | .735 |
|  | RLA | .39 | .999 | .29 | .999 | | -.06 | .999 |
|  | ELA | .11 | .999 | .16 | .999 | | .13 | .999 |
|  | BML | .04 | .999 | .04 | .999 | | .11 | .999 |
|  | RLA*Age | .08 | .999 | .18 | .999 | | .53 | .999 |
|  | ELA*Age | .21 | .999 | .14 | .999 | | .18 | .999 |
|  | BML*Age | -.02 | .999 | -.02 | .999 | | -.11 | .999 |
|  | *R*^2^ | .44 | | .43 | | | .35 | |
|  | Δ*R*^2^ | .00 | | .00 | | | .01 | |

*Note.* Sex: -1 = male, 1 = female; SES = socioeconomic status: -1 = no postsecondary maternal education, 1 = postsecondary maternal education; BML = bi/multilingualism: -1 = yes, 1 = no; RLA = receptive language ability; ELA = expressive language ability; *p*_H_ = *p* value adjusted with the correction by Hommel (1988). Significant results are presented in bold.

****p*_H_ < .001.

**Table S21**

*Post-Hoc Hierarchical Regression Analyses for Sex, SES, Age, Receptive Language Ability, Expressive Language Ability, and Bi/Multilingualism as Predictors and Moderator (Age) of Scores on the Seven Group Factors of the Intelligence Domain From the Intelligence and Development Scales–2*

| Step | Predictor | Visual Processing | | Processing Speed | | Auditory Short-Term Memory | | | Visuospatial Short-Term Memory | | Abstract Reasoning | | Verbal Reasoning | | Long-Term Memory | |
| --- | --- | --- | --- | --- | --- | --- | --- | --- | --- | --- | --- | --- | --- | --- | --- | --- |
|  |  | β | *p*_H_ | β | *p*_H_ | β | | *p*_H_ | β | *p*_H_ | β | *p*_H_ | β | *p*_H_ | β | *p*_H_ |
| 1 | Sex | -.13 | .147 | .05 | .999 | .05 | | .999 | .03 | .999 | .08 | .999 | .04 | .999 | .07 | .999 |
|  | SES | .19 | **< .001** | .12 | .558 | .21 | | **< .001** | .13 | .181 | .20 | **< .001** | .23 | **< .001** | .13 | .286 |
|  | Age | .00 | .999 | -.01 | .999 | -.01 | | .999 | -.00 | .999 | -.04 | .999 | -.00 | .999 | .04 | .999 |
|  | *R*^2^ | .05 | | .02 | | .05 | | | .02 | | .05 | | .06 | | .02 | |
| 2 | Sex | -.17 | **< .001** | .01 | .999 | -.00 | | .999 | -.01 | .999 | .03 | .999 | -.02 | .999 | .02 | .999 |
|  | SES | .14 | **.026** | .07 | .999 | .15 | | **.005** | .08 | .999 | .14 | **.015** | .15 | **< .001** | .06 | .999 |
|  | Age | .00 | .999 | -.01 | .999 | -.01 | | .999 | -.00 | .999 | -.04 | .999 | -.00 | .999 | .04 | .999 |
|  | RLA | .35 | **< .001** | .32 | **< .001** | .40 | | **< .001** | .33 | **< .001** | .39 | **< .001** | .55 | **< .001** | .40 | **< .001** |
|  | *R*^2^ | .17 | | .12 | | .20 | | | .12 | | .19 | | .34 | | .17 | |
|  | Δ*R*^2^ | **.12***** | | **.10***** | | **.15***** | | | **.10***** | | **.14***** | | **.28***** | | **.15***** | |
| 3 | Sex | -.18 | **< .001** | .00 | .999 | -.01 | | .999 | -.01 | .999 | .02 | .999 | -.04 | .999 | .01 | .999 |
|  | SES | .12 | .154 | .06 | .999 | .13 | | .055 | .07 | .999 | .12 | .133 | .12 | **.026** | .05 | .999 |
|  | Age | .00 | .999 | -.01 | .999 | -.00 | | .999 | -.00 | .999 | -.04 | .999 | .00 | .999 | .05 | .999 |
|  | RLA | .27 | **< .001** | .27 | **< .001** | .30 | | **< .001** | .27 | **< .001** | .30 | **< .001** | .41 | **< .001** | .32 | **< .001** |
|  | ELA | .17 | **.003** | .11 | .999 | .23 | | **< .001** | .13 | .282 | .20 | **< .001** | .32 | **< .001** | .17 | **.003** |
|  | *R*^2^ | .19 | | .13 | | .24 | | | .13 | | .23 | | .42 | | .20 | |
|  | Δ*R*^2^ | **.02**** | | .01 | | **.04***** | | | .01 | | **.04***** | | **.08***** | | **.03**** | |
| 4 | Sex | -.18 | **< .001** | .00 | .999 | -.01 | | .999 | -.01 | .999 | .02 | .999 | -.04 | .999 | .01 | .999 |
|  | SES | .12 | .225 | .05 | .999 | .13 | | .058 | .06 | .999 | .12 | .199 | .13 | **.005** | .06 | .999 |
|  | Age | .00 | .999 | -.01 | .999 | -.00 | | .999 | -.00 | .999 | -.04 | .999 | -.00 | .999 | .04 | .999 |
|  | RLA | .28 | **< .001** | .28 | **< .001** | .30 | | **< .001** | .27 | **< .001** | .31 | **< .001** | .40 | **< .001** | .31 | **< .001** |
|  | ELA | .18 | **.002** | .12 | .999 | .23 | | **< .001** | .14 | .267 | .21 | **< .001** | .30 | **< .001** | .15 | **.038** |
|  | BML | -.03 | .999 | -.04 | .999 | .00 | | .999 | -.01 | .999 | -.03 | .999 | .10 | .150 | .10 | .965 |
|  | *R*^2^ | .19 | | .13 | | .24 | | | .13 | | .23 | | .43 | | .21 | |
|  | Δ*R*^2^ | .00 | | .00 | | .00 | | | .00 | | .00 | | .01 | | .01 | |
| 5 | Sex | -.18 | **< .001** | .00 | .999 | -.01 | .999 | | -.01 | .999 | .02 | .999 | -.04 | .999 | .01 | .999 |
|  | SES | .12 | .227 | .06 | .999 | .13 | .071 | | .06 | .999 | .12 | .180 | .12 | **.007** | .06 | .999 |
|  | Age | -.25 | .999 | .11 | .999 | -.03 | .999 | | -.18 | .999 | -.34 | .999 | -.08 | .999 | -.24 | .999 |
|  | RLA | .19 | .999 | .36 | .999 | .39 | .999 | | .18 | .999 | .08 | .999 | .43 | .999 | .09 | .999 |
|  | ELA | -.11 | .999 | .21 | .999 | .10 | .999 | | -.03 | .999 | .00 | .999 | .14 | .999 | -.03 | .999 |
|  | BML | -.15 | .999 | -.28 | .999 | .13 | .999 | | -.13 | .999 | -.04 | .999 | .25 | .999 | .37 | .999 |
|  | RLA*Age | .11 | .999 | -.10 | .999 | -.11 | .999 | | .11 | .999 | .28 | .999 | -.04 | .999 | .27 | .999 |
|  | ELA*Age | .35 | .999 | -.11 | .999 | .15 | .999 | | .20 | .999 | .25 | .999 | .19 | .999 | .22 | .999 |
|  | BML*Age | .12 | .999 | .24 | .999 | -.13 | .999 | | .12 | .999 | .01 | .999 | -.15 | .999 | -.27 | .999 |
|  | *R*^2^ | .20 | | .13 | | .24 | | | .14 | | .23 | | .43 | | .21 | |
|  | Δ*R*^2^ | .01 | | .00 | | .00 | | | .01 | | .00 | | .00 | | .00 | |

*Note.* Sex: -1 = male, 1 = female; SES = socioeconomic status: -1 = no postsecondary maternal education, 1 = postsecondary maternal education; BML = bi/multilingualism: -1 = yes, 1 = no; RLA = receptive language ability; ELA = expressive language ability. *p*_H_ = *p* value adjusted with the correction by Hommel (1988). Significant results are presented in bold.

***p*_H_ < .01. ****p*_H_ < .001.

**Table S22**

*Post-Hoc Hierarchical Regression Analyses for Sex, SES, Age, Receptive Language Ability, Expressive Language Ability, and Bi/Multilingualism as Predictors and Moderator (Age) of Scores on the Seven Subtests of the Intelligence Domain (Part 1) From the Intelligence and Development Scales–2*

| Step | Predictor | Shape Design | | Parrots | | | Digit and Letter Span | | | Shape Memory | | Matrices: Completion | | | Naming Categories | | | Story Recall | | | |
| --- | --- | --- | --- | --- | --- | --- | --- | --- | --- | --- | --- | --- | --- | --- | --- | --- | --- | --- | --- | --- | --- |
|  |  | β | *p*_H_ | β | | *p*_H_ | β | | *p*_H_ | β | *p*_H_ | β | | *p*_H_ | β | | *p*_H_ | Β | | *p*_H_ |  |
| 1 | Sex | -.18 | **< .001** | .05 | | .999 | .05 | | .999 | .02 | .999 | .02 | | .999 | .03 | | .999 | -.02 | | .999 |  |
|  | SES | .21 | **< .001** | .12 | | .519 | .23 | | **< .001** | .12 | .617 | .17 | | **.002** | .18 | | **< .001** | .15 | | **.017** |  |
|  | Age | -.01 | .999 | -.01 | | .999 | -.02 | | .999 | -.03 | .999 | -.04 | | .999 | .04 | | .999 | .00 | | .999 |  |
|  | *R*^2^ | .07 | | .02 | | | .06 | | | .02 | | .03 | | | .04 | | | .02 | | | |
| 2 | Sex | -.23 | **< .001** | .02 | | .999 | .00 | | .999 | -.01 | .999 | -.02 | | .999 | -.03 | | .999 | -.07 | | .999 |  |
|  | SES | .15 | **.005** | .07 | | .999 | .17 | | **< .001** | .07 | .999 | .11 | | .630 | .11 | | .511 | .08 | | .999 |  |
|  | Age | -.00 | .999 | -.01 | | .999 | -.02 | | .999 | -.03 | .999 | -.04 | | .999 | .04 | | .999 | .00 | | .999 |  |
|  | RLA | .38 | **< .001** | .30 | | **< .001** | .38 | | **< .001** | .27 | **< .001** | .35 | | **< .001** | .47 | | **< .001** | .42 | | **< .001** |  |
|  | *R*^2^ | .21 | | .10 | | | .20 | | | .08 | | .15 | | | .25 | | | .20 | | | |
|  | Δ*R*^2^ | **.14***** | | **.08***** | | | **.14***** | | | **.06***** | | **.12***** | | | **.21***** | | | **.18***** | | | |
| 3 | Sex | -.24 | **< .001** | .01 | | .999 | -.01 | | .999 | -.01 | .999 | -.03 | | .999 | -.04 | | .999 | -.09 | | .999 |  |
|  | SES | .13 | **.028** | .06 | | .999 | .15 | | **.004** | .07 | .999 | .09 | | .999 | .08 | | .999 | .06 | | .999 |  |
|  | Age | -.00 | .999 | -.00 | | .999 | -.01 | | .999 | -.03 | .999 | -.04 | | .999 | .05 | | .999 | .01 | | .999 |  |
|  | RLA | .32 | **< .001** | .24 | | **< .001** | .29 | | **< .001** | .23 | **< .001** | .26 | | **< .001** | .36 | | **< .001** | .32 | | **< .001** |  |
|  | ELA | .14 | .059 | .13 | | .366 | .20 | | **< .001** | .08 | .999 | .21 | | **< .001** | .26 | | **< .001** | .25 | | **< .001** |  |
|  | *R*^2^ | .23 | | .11 | | | .23 | | | .09 | | .18 | | | .30 | | | .24 | | | |
|  | Δ*R*^2^ | .02 | | .01 | | | **.03***** | | | .01 | | **.03***** | | | **.05***** | | | **.04***** | | | |
| 4 | Sex | -.24 | **< .001** | .01 | | .999 | -.01 | | .999 | -.01 | .999 | -.03 | | .999 | -.04 | | .999 | -.09 | | .999 |  |
|  | SES | .13 | .052 | .06 | | .999 | .15 | | **.004** | .07 | .999 | .09 | | .999 | .08 | | .999 | .07 | | .999 |  |
|  | Age | -.00 | .999 | -.00 | | .999 | -.01 | | .999 | -.03 | .999 | -.04 | | .999 | .04 | | .999 | .00 | | .999 |  |
|  | RLA | .33 | **< .001** | .24 | | **< .001** | .29 | | **< .001** | .23 | **< .001** | .26 | | **< .001** | .35 | | **< .001** | .30 | | **< .001** |  |
|  | ELA | .15 | **.025** | .13 | | .354 | .20 | | **< .001** | .09 | .999 | .21 | | **< .001** | .26 | | **< .001** | .23 | | **< .001** |  |
|  | BML | -.05 | .999 | -.01 | | .999 | .01 | | .999 | -.00 | .999 | -.02 | | .999 | .02 | | .999 | .13 | | .054 |  |
|  | *R*^2^ | .23 | | .11 | | | .23 | | | .09 | | .18 | | | .30 | | | .26 | | | |
|  | Δ*R*^2^ | .00 | | .00 | | | .00 | | | .00 | | .00 | | | .00 | | | .02 | | | |
| 5 | Sex | -.24 | **< .001** | .01 | .999 | | -.01 | .999 | | -.01 | .999 | -.03 | .999 | | -.04 | .999 | | -.09 | .999 | | |
|  | SES | .13 | .051 | .06 | .999 | | .15 | **.004** | | .07 | .999 | .09 | .999 | | .09 | .999 | | .07 | .999 | | |
|  | Age | -.17 | .999 | .12 | .999 | | -.04 | .999 | | -.25 | .999 | -.39 | .999 | | -.27 | .999 | | -.09 | .999 | | |
|  | RLA | .32 | .999 | .25 | .999 | | .35 | .999 | | .21 | .999 | -.16 | .999 | | .06 | .999 | | .16 | .999 | | |
|  | ELA | -.09 | .999 | .29 | .999 | | .10 | .999 | | -.23 | .999 | .13 | .999 | | .09 | .999 | | .25 | .999 | | |
|  | BML | -.27 | .999 | -.16 | .999 | | .16 | .999 | | -.15 | .999 | .03 | .999 | | .15 | .999 | | .44 | .999 | | |
|  | RLA*Age | .01 | .999 | -.02 | .999 | | -.07 | .999 | | .02 | .999 | .52 | .999 | | .36 | .999 | | .18 | .999 | | |
|  | ELA*Age | .29 | .999 | -.19 | .999 | | .12 | .999 | | .39 | .999 | .10 | .999 | | .20 | .999 | | -.03 | .999 | | |
|  | BML*Age | .23 | .999 | .15 | .999 | | -.15 | .999 | | .15 | .999 | -.05 | .999 | | -.12 | .999 | | -.32 | .999 | | |
|  | *R*^2^ | .24 | | .12 | | | .23 | | | .10 | | .19 | | | .30 | | | .26 | | | |
|  | Δ*R*^2^ | .01 | | .01 | | | .00 | | | .01 | | .01 | | | .00 | | | .00 | | | |

*Note.* Sex: -1 = male, 1 = female; SES = socioeconomic status: -1 = no postsecondary maternal education, 1 = postsecondary maternal education; BML = bi/multilingualism: -1 = yes, 1 = no; RLA = receptive language ability; ELA = expressive language ability; *p*_H_ = *p* value adjusted with the correction by Hommel (1988). Significant results are presented in bold.

****p*_H_ < .001.

**Table S23**

*Post-Hoc Hierarchical Regression Analyses for Sex, SES, Age, Receptive Language Ability, Expressive Language Ability, and Bi/Multilingualism as Predictors and Moderator (Age) of Scores on the Seven Subtests of the Intelligence Domain (Part 2) From the Intelligence and Development Scales–2*

| Step | Predictor | Washer Design | | Boxes | | Mixed Digit and Letter Span | | | Rotated Shape Memory | | Matrices: Odd One Out | | | Naming Opposites | | | Picture Recall | | | |
| --- | --- | --- | --- | --- | --- | --- | --- | --- | --- | --- | --- | --- | --- | --- | --- | --- | --- | --- | --- | --- |
|  |  | β | *p*_H_ | β | *p*_H_ | β | *p*_H_ | | β | *p*_H_ | β | | *p*_H_ | β | | *p*_H_ | β | | *p*_H_ |  |
| 1 | Sex | -.03 | .999 | .03 | .999 | .04 | .999 | | .04 | .999 | .12 | | .594 | .05 | | .999 | .14 | | .085 |  |
|  | SES | .11 | .999 | .10 | .999 | .17 | **.002** | | .10 | .999 | .17 | | **.001** | .23 | | **< .001** | .05 | | .999 |  |
|  | Age | -.01 | .999 | -.01 | .999 | .00 | .999 | | .03 | .999 | -.03 | | .999 | -.05 | | .999 | .00 | | .999 |  |
|  | *R*^2^ | .01 | | .01 | | .03 | | | .01 | | .04 | | | .06 | | | .02 | | | |
| 2 | Sex | -.05 | .999 | -.00 | .999 | -.00 | .999 | | .01 | .999 | .08 | | .999 | -.01 | | .999 | .11 | | .888 |  |
|  | SES | .08 | .999 | .06 | .999 | .11 | .812 | | .05 | .999 | .12 | | .248 | .15 | | **.001** | .02 | | .999 |  |
|  | Age | -.01 | .999 | -.01 | .999 | .00 | .999 | | .03 | .999 | -.03 | | .999 | -.05 | | .999 | .00 | | .999 |  |
|  | RLA | .20 | **< .001** | .26 | **< .001** | .37 | **< .001** | | .30 | **< .001** | .30 | | **< .001** | .50 | | **< .001** | .21 | | **< .001** |  |
|  | *R*^2^ | .05 | | .08 | | .16 | | | .10 | | .13 | | | .30 | | | .06 | | | |
|  | Δ*R*^2^ | **.04***** | | **.07***** | | **.13***** | | | **.09***** | | **.09***** | | | **.24***** | | | **.04***** | | | |
| 3 | Sex | -.06 | .999 | -.01 | .999 | -.01 | .999 | | -.00 | .999 | .07 | | .999 | -.03 | | .999 | .11 | | .958 |  |
|  | SES | .06 | .999 | .05 | .999 | .09 | .999 | | .04 | .999 | .11 | | .764 | .12 | | **.034** | .01 | | .999 |  |
|  | Age | -.00 | .999 | -.01 | .999 | .00 | .999 | | .03 | .999 | -.03 | | .999 | -.04 | | .999 | .00 | | .999 |  |
|  | RLA | .13 | .426 | .24 | **< .001** | .28 | **< .001** | | .23 | **< .001** | .25 | | **< .001** | .37 | | **< .001** | .20 | | **< .001** |  |
|  | ELA | .14 | .260 | .06 | .999 | .21 | **< .001** | | .16 | **.014** | .12 | | .683 | .30 | | **< .001** | .02 | | .999 |  |
|  | *R*^2^ | .06 | | .08 | | .20 | | | .12 | | .14 | | | .37 | | | .07 | | | |
|  | Δ*R*^2^ | .01 | | .00 | | **.04***** | | | **.02*** | | .01 | | | **.07***** | | | .01 | | | |
| 4 | Sex | -.06 | .999 | -.01 | .999 | -.01 | .999 | | -.00 | .999 | .07 | | .999 | -.03 | | .999 | .11 | | .989 |  |
|  | SES | .06 | .999 | .05 | .999 | .09 | .999 | | .04 | .999 | .11 | | .999 | .13 | | **.003** | .02 | | .999 |  |
|  | Age | -.00 | .999 | -.01 | .999 | .00 | .999 | | .03 | .999 | -.03 | | .999 | -.05 | | .999 | .00 | | .999 |  |
|  | RLA | .14 | .452 | .24 | **< .001** | .28 | **< .001** | | .24 | **< .001** | .25 | | **< .001** | .35 | | **< .001** | .20 | | **< .001** |  |
|  | ELA | .14 | .302 | .07 | .999 | .21 | **< .001** | | .17 | **.011** | .13 | | .461 | .28 | | **< .001** | .02 | | .999 |  |
|  | BML | -.00 | .999 | -.05 | .999 | -.02 | .999 | | -.03 | .999 | -.04 | | .999 | .16 | | **< .001** | .03 | | .999 |  |
|  | *R*^2^ | .06 | | .08 | | .20 | | | .12 | | .15 | | | .40 | | | .07 | | | |
|  | Δ*R*^2^ | .00 | | .00 | | .00 | | | .00 | | .01 | | | **.03***** | | | .00 | | | |
| 5 | Sex | -.06 | .999 | -.01 | .999 | -.01 | | .999 | -.00 | .999 | .08 | .999 | | -.03 | .999 | | .11 | .877 | | |
|  | SES | .06 | .999 | .05 | .999 | .08 | | .999 | .04 | .999 | .11 | .999 | | .13 | **.004** | | .02 | .999 | | |
|  | Age | -.29 | .999 | .07 | .999 | -.10 | | .999 | -.12 | .999 | -.21 | .999 | | .10 | .999 | | -.46 | .999 | | |
|  | RLA | -.01 | .999 | .35 | .999 | .31 | | .999 | .02 | .999 | .29 | .999 | | .69 | **.010** | | -.07 | .999 | | |
|  | ELA | -.13 | .999 | .07 | .999 | .03 | | .999 | .17 | .999 | -.18 | .999 | | .14 | .999 | | -.37 | .999 | | |
|  | BML | .04 | .999 | -.34 | .999 | .08 | | .999 | -.05 | .999 | -.08 | .999 | | .29 | .999 | | .13 | .999 | | |
|  | RLA*Age | .18 | .999 | -.14 | .999 | -.04 | | .999 | .27 | .999 | -.05 | .999 | | -.42 | .999 | | .34 | .999 | | |
|  | ELA*Age | .33 | .999 | .01 | .999 | .23 | | .999 | -.01 | .999 | .38 | .999 | | .17 | .999 | | .47 | .999 | | |
|  | BML*Age | -.05 | .999 | .29 | .999 | -.11 | | .999 | .02 | .999 | .05 | .999 | | -.14 | .999 | | -.10 | .999 | | |
|  | *R*^2^ | .07 | | .09 | | .20 | | | .13 | | .15 | | | .40 | | | .08 | | | |
|  | Δ*R*^2^ | .01 | | .01 | | .00 | | | .01 | | .00 | | | .00 | | | .01 | | | |

*Note.* Sex: -1 = male, 1 = female; SES = socioeconomic status: -1 = no postsecondary maternal education, 1 = postsecondary maternal education; BML = bi/multilingualism: -1 = yes, 1 = no; RLA = receptive language ability; ELA = expressive language ability; *p*_H_ = *p* value adjusted with the correction by Hommel (1988). Significant results are presented in bold.

**p*_H_ < .05. ****p*_H_ < .001.

**Table S24**

*Post-Hoc Hierarchical Regression Analyses for Sex, SES, Age, Receptive Language Ability, Expressive Language Ability, and Bi/Multilingualism as Predictors and Moderator (Age) of Scores on the Composite and Subtests of the Executive Functions Domain From the Intelligence and Development Scales–2*

| Step | Predictor | Executive functions composite | | | Listing Words | | | Divided Attention | | | Animal Colors | | | Drawing Routes | | | |
| --- | --- | --- | --- | --- | --- | --- | --- | --- | --- | --- | --- | --- | --- | --- | --- | --- | --- |
|  |  | β | | *p*_H_ | β | | *p*_H_ | β | | *p*_H_ | β | *p*_H_ | | β | | *p*_H_ |  |
| 1 | Sex | .09 | | .999 | .14 | | .072 | .05 | | .999 | .09 | .999 | | -.05 | | .999 |  |
|  | SES | .17 | | **.001** | .16 | | **.008** | .13 | | .255 | .06 | .999 | | .13 | | .263 |  |
|  | Age | .03 | | .999 | -.03 | | .999 | .02 | | .999 | .09 | .999 | | -.00 | | .999 |  |
|  | *R*^2^ | .04 | | | .05 | | | .02 | | | .02 | | | .02 | | | |
| 2 | Sex | .04 | | .999 | .09 | | .999 | .01 | | .999 | .06 | .999 | | -.08 | | .999 |  |
|  | SES | .11 | | .683 | .10 | | .999 | .07 | | .999 | .03 | .999 | | .10 | | .999 |  |
|  | Age | .03 | | .999 | -.02 | | .999 | .02 | | .999 | .09 | .999 | | -.00 | | .999 |  |
|  | RLA | .42 | | **< .001** | .38 | | **< .001** | .37 | | **< .001** | .23 | **< .001** | | .18 | | **< .001** |  |
|  | *R*^2^ | .21 | | | .18 | | | .15 | | | .07 | | | .05 | | | |
|  | Δ*R*^2^ | **.17***** | | | **.13***** | | | **.13***** | | | **.05***** | | | **.03***** | | | |
| 3 | Sex | .03 | | .999 | .08 | | .999 | .00 | | .999 | .06 | .999 | | -.08 | | .999 |  |
|  | SES | .09 | | .999 | .09 | | .999 | .06 | | .999 | .02 | .999 | | .09 | | .999 |  |
|  | Age | .03 | | .999 | -.02 | | .999 | .02 | | .999 | .09 | .999 | | -.00 | | .999 |  |
|  | RLA | .33 | | **< .001** | .31 | | **< .001** | .29 | | **< .001** | .19 | **< .001** | | .13 | | .834 |  |
|  | ELA | .20 | | **< .001** | .16 | | **.018** | .19 | | **< .001** | .10 | .999 | | .13 | | .920 |  |
|  | *R*^2^ | .24 | | | .20 | | | .18 | | | .08 | | | .06 | | | |
|  | Δ*R*^2^ | **.03***** | | | **.02*** | | | **.03***** | | | .01 | | | .01 | | | |
| 4 | Sex | .03 | | .999 | .08 | | .999 | .00 | | .999 | .06 | .999 | | -.08 | | .999 |  |
|  | SES | .09 | | .999 | .09 | | .999 | .06 | | .999 | .01 | .999 | | .09 | | .999 |  |
|  | Age | .03 | | .999 | -.02 | | .999 | .02 | | .999 | .09 | .999 | | -.00 | | .999 |  |
|  | RLA | .33 | | **< .001** | .31 | | **< .001** | .28 | | **< .001** | .21 | **< .001** | | .13 | | .849 |  |
|  | ELA | .21 | | **< .001** | .15 | | **.036** | .18 | | **.001** | .11 | .999 | | .13 | | .945 |  |
|  | BML | -.02 | | .999 | .02 | | .999 | .05 | | .999 | -.10 | .999 | | -.01 | | .999 |  |
|  | *R*^2^ | .24 | | | .20 | | | .18 | | | .09 | | | .06 | | | |
|  | Δ*R*^2^ | .00 | | | .00 | | | .00 | | | .01 | | | .00 | | | |
| 5 | Sex | .03 | .999 | | .09 | .999 | | .01 | .999 | | .06 | | .999 | -.08 | .999 | | |
|  | SES | .08 | .999 | | .08 | .999 | | .06 | .999 | | .01 | | .999 | .09 | .999 | | |
|  | Age | -.30 | .999 | | -.37 | .999 | | -.29 | .999 | | -.12 | | .999 | -.03 | .999 | | |
|  | RLA | .40 | .999 | | .30 | .999 | | .36 | .999 | | .26 | | .999 | .22 | .999 | | |
|  | ELA | -.34 | .999 | | -.35 | .999 | | -.34 | .999 | | -.25 | | .999 | -.01 | .999 | | |
|  | BML | .09 | .999 | | .47 | .999 | | .16 | .999 | | -.27 | | .999 | -.11 | .999 | | |
|  | RLA*Age | -.09 | .999 | | -.00 | .999 | | -.10 | .999 | | -.08 | | .999 | -.12 | .999 | | |
|  | ELA*Age | .68 | .845 | | .62 | .999 | | .64 | .999 | | .45 | | .999 | .17 | .999 | | |
|  | BML*Age | -.11 | .999 | | -.46 | .999 | | -.12 | .999 | | .17 | | .999 | .11 | .999 | | |
|  | *R*^2^ | .25 | | | .22 | | | .19 | | | .09 | | | .06 | | | |
|  | Δ*R*^2^ | .01 | | | .02 | | | .01 | | | .00 | | | .00 | | | |

*Note.* Sex: -1 = male, 1 = female; SES = socioeconomic status: -1 = no postsecondary maternal education, 1 = postsecondary maternal education; BML = bi/multilingualism: -1 = yes, 1 = no; RLA = receptive language ability; ELA = expressive language ability; *p*_H_ = *p* value adjusted with the correction by Hommel (1988). Significant results are presented in bold.

**p*_H_ < .05. ****p*_H_ < .001.

**Table S25**

*Post-Hoc Hierarchical Regression Analyses for Sex, SES, Age, Receptive Language Ability, Expressive Language Ability, and Bi/Multilingualism as Predictors and Moderator (Age) of Scores on the Composite and Subtests of the Psychomotor Skills Domain From the Intelligence and Development Scales–2*

| Step | Predictor | Psychomotor skills composite | | | Gross Motor Skills | | | Fine Motor Skills | | Visuomotor Skills | | |
| --- | --- | --- | --- | --- | --- | --- | --- | --- | --- | --- | --- | --- |
|  |  | β | *p*_H_ | | β | *p*_H_ | | β | *p*_H_ | β | | *p*_H_ |
| 1 | Sex | .12 | .634 | | .00 | .999 | | .18 | **< .001** | .09 | | .999 |
|  | SES | .08 | .999 | | .05 | .999 | | .02 | .999 | .12 | | .685 |
|  | Age | .03 | .999 | | .04 | .999 | | .02 | .999 | .00 | | .999 |
|  | *R*^2^ | .02 | | | .00 | | | .03 | | .02 | | |
| 2 | Sex | .08 | .999 | | -.02 | .999 | | .16 | **.005** | .06 | | .999 |
|  | SES | .03 | .999 | | .01 | .999 | | -.02 | .999 | .07 | | .999 |
|  | Age | .03 | .999 | | .04 | .999 | | .03 | .999 | .01 | | .999 |
|  | RLA | .33 | **< .001** | | .23 | **< .001** | | .23 | **< .001** | .28 | | **< .001** |
|  | *R*^2^ | .13 | | | .05 | | | .09 | | .10 | | |
|  | Δ*R*^2^ | **.11***** | | | **.05***** | | | **.06***** | | **.08***** | | |
| 3 | Sex | .07 | .999 | | -.03 | .999 | | .15 | **.010** | .06 | | .999 |
|  | SES | .01 | .999 | | -.00 | .999 | | -.03 | .999 | .06 | | .999 |
|  | Age | .03 | .999 | | .04 | .999 | | .03 | .999 | .01 | | .999 |
|  | RLA | .27 | **< .001** | | .18 | **.002** | | .19 | **< .001** | .22 | | **< .001** |
|  | ELA | .15 | **.022** | | .11 | .999 | | .09 | .999 | .13 | | .275 |
|  | *R*^2^ | .15 | | | .06 | | | .09 | | .11 | | |
|  | Δ*R*^2^ | **.02*** | | | .01 | | | .00 | | .01 | | |
| 4 | Sex | .07 | .999 | | -.03 | .999 | | .15 | **.010** | .06 | | .999 |
|  | SES | .01 | .999 | | -.00 | .999 | | -.03 | .999 | .05 | | .999 |
|  | Age | .03 | .999 | | .04 | .999 | | .03 | .999 | .01 | | .999 |
|  | RLA | .27 | **< .001** | | .19 | **.002** | | .19 | **< .001** | .23 | | **< .001** |
|  | ELA | .16 | **.010** | | .11 | .999 | | .10 | .999 | .15 | | .097 |
|  | BML | -.05 | .999 | | -.03 | .999 | | -.01 | .999 | -.07 | | .999 |
|  | *R*^2^ | .15 | | | .06 | | | .09 | | .12 | | |
|  | Δ*R*^2^ | .00 | | | .00 | | | .00 | | .01 | | |
| 5 | Sex | .07 | | .999 | -.03 | | .999 | .15 | **.007** | .06 | .999 | |
|  | SES | .01 | | .999 | -.01 | | .999 | -.03 | .999 | .05 | .999 | |
|  | Age | -.22 | | .999 | -.19 | | .999 | -.25 | .999 | .04 | .999 | |
|  | RLA | .26 | | .999 | .19 | | .999 | .16 | .999 | .29 | .999 | |
|  | ELA | -.20 | | .999 | -.23 | | .999 | -.28 | .999 | .13 | .999 | |
|  | BML | .08 | | .999 | .22 | | .999 | .21 | .999 | -.37 | .999 | |
|  | RLA*Age | .01 | | .999 | -.01 | | .999 | .04 | .999 | -.07 | .999 | |
|  | ELA*Age | .44 | | .999 | .43 | | .999 | .45 | .999 | .01 | .999 | |
|  | BML*Age | -.13 | | .999 | -.26 | | .999 | -.22 | .999 | .31 | .999 | |
|  | *R*^2^ | .15 | | | .07 | | | .10 | | .12 | | |
|  | Δ*R*^2^ | .00 | | | .01 | | | .01 | | .00 | | |

*Note.* Sex: -1 = male, 1 = female; SES = socioeconomic status: -1 = no postsecondary maternal education, 1 = postsecondary maternal education; BML = bi/multilingualism: -1 = yes, 1 = no; RLA = receptive language ability; ELA = expressive language ability; *p*_H_ = *p* value adjusted with the correction by Hommel (1988). Significant results are presented in bold.

**p*_H_ < .05. ****p*_H_ < .001.

**Table S26**

*Post-Hoc Hierarchical Regression Analyses for Sex, SES, Age, Receptive Language Ability, Expressive Language Ability, and Bi/Multilingualism as Predictors and Moderator (Age) of Scores on the Composite and Subtests of the Social-Emotional Skills Domain From the Intelligence and Development Scales–2*

| Step | Predictor | Social-emotional skills composite | | | Identifying Emotions | | Regulating Emotions | | | Socially Competent Behavior | | |
| --- | --- | --- | --- | --- | --- | --- | --- | --- | --- | --- | --- | --- |
|  |  | β | | *p*_H_ | β | *p*_H_ | β | | *p*_H_ | β | | *p*_H_ |
| 1 | Sex | .07 | | .999 | .06 | .999 | .04 | | .999 | .05 | | .999 |
|  | SES | .07 | | .999 | .09 | .999 | .03 | | .999 | .03 | | .999 |
|  | Age | .06 | | .999 | .07 | .999 | .00 | | .999 | .05 | | .999 |
|  | *R*^2^ | .01 | | | .02 | | .00 | | | .01 | | |
| 2 | Sex | .04 | | .999 | .03 | .999 | .02 | | .999 | .04 | | .999 |
|  | SES | .03 | | .999 | .06 | .999 | -.00 | | .999 | .02 | | .999 |
|  | Age | .06 | | .999 | .07 | .999 | .00 | | .999 | .05 | | .999 |
|  | RLA | .21 | | **< .001** | .19 | **< .001** | .17 | | **< .001** | .11 | | .999 |
|  | *R*^2^ | .06 | | | .05 | | .03 | | | .02 | | |
|  | Δ*R*^2^ | **.05***** | | | **.03***** | | **.03***** | | | .01 | | |
| 3 | Sex | .03 | | .999 | .03 | .999 | .01 | | .999 | .04 | | .999 |
|  | SES | .02 | | .999 | .05 | .999 | -.02 | | .999 | .01 | | .999 |
|  | Age | .06 | | .999 | .07 | .999 | .00 | | .999 | .05 | | .999 |
|  | RLA | .14 | | .262 | .14 | .200 | .10 | | .999 | .06 | | .999 |
|  | ELA | .18 | | **.004** | .12 | .989 | .17 | | **.013** | .10 | | .999 |
|  | *R*^2^ | .08 | | | .06 | | .05 | | | .02 | | |
|  | Δ*R*^2^ | **.02**** | | | .01 | | **.02*** | | | .00 | | |
| 4 | Sex | .03 | | .999 | .03 | .999 | .01 | | .999 | .04 | | .999 |
|  | SES | .02 | | .999 | .05 | .999 | -.02 | | .999 | .00 | | .999 |
|  | Age | .06 | | .999 | .07 | .999 | .00 | | .999 | .05 | | .999 |
|  | RLA | .14 | | .209 | .14 | .205 | .10 | | .999 | .07 | | .999 |
|  | ELA | .18 | | **.003** | .12 | .999 | .17 | | **.017** | .11 | | .999 |
|  | BML | -.03 | | .999 | -.01 | .999 | -.00 | | .999 | -.05 | | .999 |
|  | *R*^2^ | .08 | | | .06 | | .05 | | | .03 | | |
|  | Δ*R*^2^ | .00 | | | .00 | | .00 | | | .01 | | |
| 5 | Sex | .04 | .999 | | .03 | .999 | .02 | .999 | | .04 | .999 | |
|  | SES | .01 | .999 | | .04 | .999 | -.02 | .999 | | .00 | .999 | |
|  | Age | .10 | .999 | | .43 | .999 | -.25 | .999 | | .06 | .999 | |
|  | RLA | .44 | .999 | | .55 | .999 | .17 | .999 | | .26 | .999 | |
|  | ELA | -.07 | .999 | | .23 | .999 | -.27 | .999 | | -.07 | .999 | |
|  | BML | .10 | .999 | | .27 | .999 | -.01 | .999 | | -.05 | .999 | |
|  | RLA*Age | -.38 | .999 | | -.51 | .999 | -.09 | .999 | | -.23 | .999 | |
|  | ELA*Age | .31 | .999 | | -.13 | .999 | .53 | .999 | | .22 | .999 | |
|  | BML*Age | -.13 | .999 | | -.29 | .999 | .01 | .999 | | -.00 | .999 | |
|  | *R*^2^ | .09 | | | .08 | | .06 | | | .03 | | |
|  | Δ*R*^2^ | .01 | | | .02 | | .01 | | | .00 | | |

*Note.* Sex: -1 = male, 1 = female; SES = socioeconomic status: -1 = no postsecondary maternal education, 1 = postsecondary maternal education; BML = bi/multilingualism: -1 = yes, 1 = no; RLA = receptive language ability; ELA = expressive language ability; *p*_H_ = *p* value adjusted with the correction by Hommel (1988). Significant results are presented in bold.

**p*_H_ < .05. ***p*_H_ < .01. ****p*_H_ < .001.

**Table S27**

*Post-Hoc Hierarchical Regression Analyses for Sex, SES, Age, Receptive Language Ability, Expressive Language Ability, and Bi/Multilingualism as Predictors and Moderator (Age) of Scores on the Subtests of the Basic Skills Domain From the Intelligence and Development Scales–2*

| Step | Predictor | Logical-Mathematical Reasoning | | Reading | | | Spelling | |
| --- | --- | --- | --- | --- | --- | --- | --- | --- |
|  |  | β | *p*_H_ | β | | *p*_H_ | β | *p*_H_ |
| 1 | Sex | -.07 | .999 | .05 | | .999 | .21 | **.001** |
|  | SES | .22 | **< .001** | .16 | | .156 | .06 | .999 |
|  | Age | -.12 | .540 | .10 | | .999 | .00 | .999 |
|  | *R*^2^ | .06 | | .04 | | | .05 | |
| 2 | Sex | -.12 | **.033** | -.00 | | .999 | .16 | .096 |
|  | SES | .14 | **.003** | .10 | | .999 | .01 | .999 |
|  | Age | -.11 | .116 | .12 | | .999 | .02 | .999 |
|  | RLA | .46 | **< .001** | .40 | | **< .001** | .36 | **< .001** |
|  | *R*^2^ | .27 | | .19 | | | .17 | |
|  | Δ*R*^2^ | **.21***** | | **.15***** | | | **.12***** | |
| 3 | Sex | -.14 | **.001** | -.02 | | .999 | .14 | .216 |
|  | SES | .11 | .090 | .06 | | .999 | -.02 | .999 |
|  | Age | -.11 | .072 | .14 | | .198 | .05 | .999 |
|  | RLA | .34 | **< .001** | .26 | | **< .001** | .23 | **< .001** |
|  | ELA | .29 | **< .001** | .31 | | **< .001** | .31 | **< .001** |
|  | *R*^2^ | .34 | | .26 | | | .25 | |
|  | Δ*R*^2^ | **.07***** | | **.07***** | | | **.08***** | |
| 4 | Sex | -.14 | **.001** | -.02 | | .999 | .14 | .217 |
|  | SES | .11 | .155 | .06 | | .999 | -.02 | .999 |
|  | Age | -.11 | .085 | .14 | | .189 | .05 | .999 |
|  | RLA | .35 | **< .001** | .27 | | **< .001** | .23 | **< .001** |
|  | ELA | .30 | **< .001** | .31 | | **< .001** | .31 | **< .001** |
|  | BML | -.04 | .999 | -.03 | | .999 | .02 | .999 |
|  | *R*^2^ | .34 | | .26 | | | .25 | |
|  | Δ*R*^2^ | .00 | | .00 | | | .00 | |
| 5 | Sex | -.14 | **.002** | -.02 | .999 | | .14 | .331 |
|  | SES | .11 | .161 | .06 | .999 | | -.02 | .999 |
|  | Age | -.29 | .999 | .10 | .999 | | -.26 | .999 |
|  | RLA | .28 | .999 | .17 | .999 | | -.34 | .999 |
|  | ELA | .10 | .999 | .31 | .999 | | .16 | .999 |
|  | BML | .00 | .999 | -.07 | .999 | | -.19 | .999 |
|  | RLA*Age | .08 | .999 | .10 | .999 | | .61 | .999 |
|  | ELA*Age | .24 | .999 | -.00 | .999 | | .15 | .999 |
|  | BML*Age | -.05 | .999 | .04 | .999 | | .21 | .999 |
|  | *R*^2^ | .34 | | .26 | | | .26 | |
|  | Δ*R*^2^ | .00 | | .00 | | | .01 | |

*Note.* Sex: -1 = male, 1 = female; SES = socioeconomic status: -1 = no postsecondary maternal education, 1 = postsecondary maternal education; BML = bi/multilingualism: -1 = yes, 1 = no; RLA = receptive language ability; ELA = expressive language ability; *p*_H_ = *p* value adjusted with the correction by Hommel (1988). The basic skills composite was not investigated since the receptive and expressive language abilities are also included in the calculation of this score. Significant results are presented in bold.

****p*_H_ < .001.

**References**

Hommel, G. (1988). A stagewise rejective multiple test procedure based on a modified Bonferroni test. *Biometrika*, *75*, 383–386.
